# Gelling and reducing agents are potential carbon and energy sources in culturing of anaerobic microorganisms

**DOI:** 10.1128/aem.02276-24

**Published:** 2025-02-12

**Authors:** Yi-Fan Liu, Liu Yang, Qing-Ping He, Yi-Lin Xu, Yu-Tong Zhu, Yan-Le Mi, Lei Zhou, Shi-Zhong Yang, Ji-Dong Gu, Bo-Zhong Mu

**Affiliations:** 1 State Key Laboratory of Bioreactor Engineering and School of Chemistry and Molecular Engineering, East China University of Science and Technology 47860 , Shanghai, China; 2 Engineering Research Center of MEOR, East China University of Science and Technology 47860 , Shanghai, China; 3 Environmental Science and Engineering Group, Guangdong Technion-Israel Institute of Technology 543072 , Shantou, Guangdong, China; Shanghai Jiao Tong University, Shanghai, China

**Keywords:** agar, gellan gum, L-cysteine, Atribacterota, oil reservoir, mangrove soil

## Abstract

The majority of microorganisms in the environment have yet to be isolated in pure cultures, and the reasons behind this phenomenon remain elusive. In this study, we investigated the possibility of the commonly used gelling agent including agar and gellan gum as a source of carbon and energy in anaerobic roll-tube cultivation from one mangrove sediment sample and two high-temperature oilfield samples. Based on growth tests and genomic evidence, anaerobic gellan degraders were retrieved from genera of *Clostridium*, *Lacrimispora*, and lineages from the rarely cultivated phylum Atribacterota. Anaerobic agarolytic microorganisms were found to be members of *Bacillus* and *Clostridium*. We also proved the role of carbon and energy sources of L-cysteine, a routine agent used to make culture media anoxic/anaerobic in both enrichment cultures and isolated strains representing *Acetomicrobium*, *Thermodesulfovibrio*, *Lacrimispora*, *Clostridium*, *Bacillus*, *Coprothermobacter*, *Citrobacter*, and *Enterobacter*. Furthermore, the isolates and enriched microbial communities utilizing L-cysteine under anaerobic conditions were mainly through L-cysteine desulfuration to pyruvate, ammonia, and sulfide. This study demonstrates that the widely used gelling and reducing agents in the basal medium can serve as carbon and energy sources for anaerobic microorganisms and thus may bias the enrichment and isolation.

**IMPORTANCE:**

Most microbial species inhabiting natural environments have not been isolated in pure cultures using conventional media and laboratory conditions, but the reason behind this is unclear. Here, we provided a new explanation for the phenomenon, in that both the gelling agents, like agar and gellan gum, and reducing agent L-cysteine-HCl in the media provide extra carbon and energy sources to microorganisms and therefore decrease the chance in isolation specifically for the supplemented substrate which is supposed to be the sole source of carbon and energy. This result demonstrated that further improvement in the effectiveness of isolation of targeted microorganisms will be facilitated by subtracting the overlooked organic ingredients in the medium and more innovations.

## INTRODUCTION

Cultivation of microorganisms in the laboratory using artificial media mimicking the environments is the most fundamental microbiological method to examine the taxonomy, morphology, ecophysiology, and metabolism of microorganisms inhabiting diverse artificial and natural ecosystems (1). Gelling agents are the major components in preparing solid culture media in agar plates and Hungate roll tubes for aerobic and anaerobic strain isolation, respectively (2). Since the initial introduction of agar in microbiology as a gelling agent in the late 19th century, it has been used routinely for over 120 years. Agar is a major component of the cell walls of red algae, which is a mixture of heterogeneous galactans, mainly containing 3,6-anhydro-L-galactoses (or L-galactose-6-sulfates), D-galactoses, and L-galactoses alternately linked by β-(1,4) and α-(1,3) linkages (3). A wide range of microorganisms have been reported to utilize agar as the sole source of energy and carbon, including species in *Bacillus*, *Pseudomonas*, *Alteromonas*, *Vibrio*, *Cytophaga*, *Agarivorans*, and *Thalassomona*s (4). These agarolytic microorganisms usually degrade agar using extracellular or intracellular agarase, which could be classified into *α*-agarase and *β*-agarase according to the cleavage pattern (4). With rare exceptions, all the discovered agarolytic microorganisms are aerobes, and only three strains of anaerobic thermophiles belonging to the genera of *Thermoanaerobacter* and *Caldoanaerobacter* with an agarolytic activity are known so far (5).

Many alternative gelling agents were discovered with time, such as carrageenan, kappa-carrageenan, and gellan gum, with gellan gum being the most widely used one for plant tissue and microorganism culture (6). Gellan gum is a bacterial polysaccharide produced by *Sphingomonas* spp. (6). The molecular structure of gellan gum is a linear heteropolysaccharide consisting of repeating units of beta-D-glucose, beta-D-glucuronic acid, beta-D-glucose, and alpha-L-rhamnose. An increasing number of studies have reported that using gellan gum as a replacement gelling agent to agar improves the culturability and diversity of microorganisms from seawater (7), freshwater sediment (8), and soils (9–11) and helps to isolate novel bacteria from previously uncultured candidate divisions from geothermal soils (12) and oil reservoirs (13). Moreover, gellan gum also significantly stimulated the colony formation of the representative slow-growing microorganism from lake sediments (14). Due to its high thermal stability, gellan gum has been often used to culture (hyper)thermophiles that grow at temperatures sufficiently high so that agar is not solidified (15). For example, several studies have observed that gellan gum improves the culturable diversity of some rare thermophilic *Actinobacteria*, several anaerobic (hyper)thermophilic marine microbes, and previously uncultured bacteria from soils (16, 17). Unlike agar, the knowledge of microbial degradation of gellan gum is scarce and currently limited to aerobic microorganisms (18). Microbial gellan degradation is facilitated by gellan lyases, secreted by species of *Bacillus*, *Sphingomonas*, and *Geobacillus* (19). For decades, the effect of gelling agents being a potential carbon and energy source on strain isolation, especially under anaerobic conditions, has not been systematically evaluated.

Moreover, reducing agents are usually amended into the media to remove oxygen and hence decrease the redox potential to allow the growth of obligate anaerobes (2). A variety of reductants have been used, including thioglycolate, L-cysteine (L-Cys), sodium sulfide, and hydrogen with a platinum catalyst. L-Cys is the most common reductant for fastidious anaerobes in clinical microbiology (20) and is often used as a supplementary reductant to sodium sulfide for strict anaerobe cultivation (21). However, L-Cys is a common substrate in many bacterial species (22), and the metabolism of L-Cys usually starts with desulfuration to pyruvate which is catalyzed by L-Cys desulfurases using pyridoxal 5′-phosphate (PLP)-based chemistry (23). Enzymes having L-Cys desulfurase activity include cystathionine β-lyase (MetC), Cys synthase A/O-acetylserine sulfhydrylase A (CysK), Cys synthase B/O-acetylserine sulfhydrylase B (CysM), β-cystathionase (MalY), tryptophanase (TNaA), and a newly discovered threonine dehydratase (psTD) (24). A recent study demonstrated that a biohybrid system composed of the anaerobic acetogenic bacterium *Moorella thermoacetica* and cadmium sulfide nanoparticles makes acetate from L-Cys independent of light, and the routine use of Cys as a reducing agent without caution in anaerobic cultivation may greatly affect and even falsify the interpretation of experiments (25).

In this study, we tested the potential that agar, gellan gum, and L-Cys could be used as electron donors (energy source and carbon source) by anaerobic microorganisms. We prepared the Hungate roll-tube medium by removing all organic substrates except for gelling agents and L-Cys and isolated anaerobes that utilize agar or gellan gum from production water from oil reservoirs and mangrove soil. We also prepared the enrichment culture medium containing only L-Cys as the electron donor to cultivate microbial communities under anoxic conditions of fermentation, sulfate reduction, and nitrate reduction.

## RESULTS

### Counts of colonies in the roll tubes

Roll tubes were prepared with either gellan gum (denoted as G) or agar (A) as the gelling agent and amended with different doses of L-Cys (see Materials and Methods for details). Three different anoxic conditions, namely, fermentative (FE), sulfate-reducing (SR), and nitrate-reducing (NR) conditions, were examined to evaluate the culturability of microorganisms from three different environmental samples, including two production water samples from Shengli and Jiangsu oilfields (denoted as SL and JS, respectively) and one Hong Kong mangrove sediment sample (HK). Altogether, the combination of different inocula and incubation conditions generated 18 experimental groups. According to the *in situ* temperatures of the environments where these inocula were sampled, the HK groups were incubated at room temperature (around 18℃–24°C), and the SL and JS groups were incubated at elevated temperatures of 55 and 64°C, respectively.

**Fig 1:**
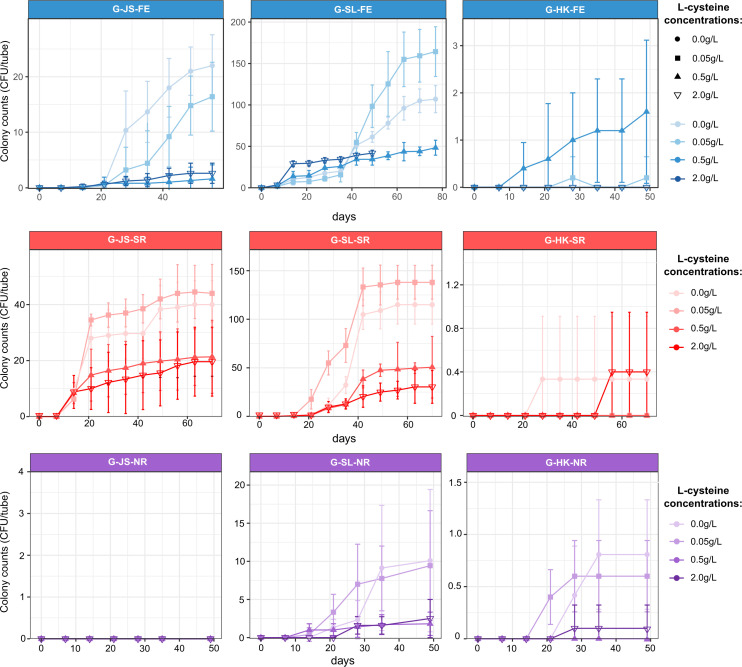
Total colony-forming units (CFU) in roll tubes using gellan gum as the gelling agent. JS and SL represent samples from Jiangsu and Shengli oilfield, respectively, and HK represents the mangrove sediment sample in Hong Kong. The roll tubes were incubated under anoxic conditions including fermentative (FE), sulfate-reducing (SR), and nitrate-reducing (NR) conditions. CFU counts are averages from ten replicate roll tubes. Error bars represent standard deviations.

In most of the tubes, the average number of colonies increased linearly after an initial lag phase (Fig. 1). Due to the relatively slow growth rates of anaerobic microorganisms, numbers of colonies were monitored over 7 to 11 weeks of incubation until saturation plateaus were reached. When using gellan gum as the gelling agent, samples inoculated with the HK mangrove sediments formed only sporadic colonies under all three conditions, indicating fewer microorganisms that could utilize gellan gum or L-Cys under anaerobic conditions in the HK sediments. Compared with the samples incubated under the other two conditions, samples incubated under the NR condition showed lower culturability regardless of the inoculum used, and especially, no colonies were detected in the Jiangsu oilfield (group G-JS-NR). These results suggested that there are relatively few nitrate-reducing microorganisms capable of utilizing constituents in gellan.

**Fig 2:**
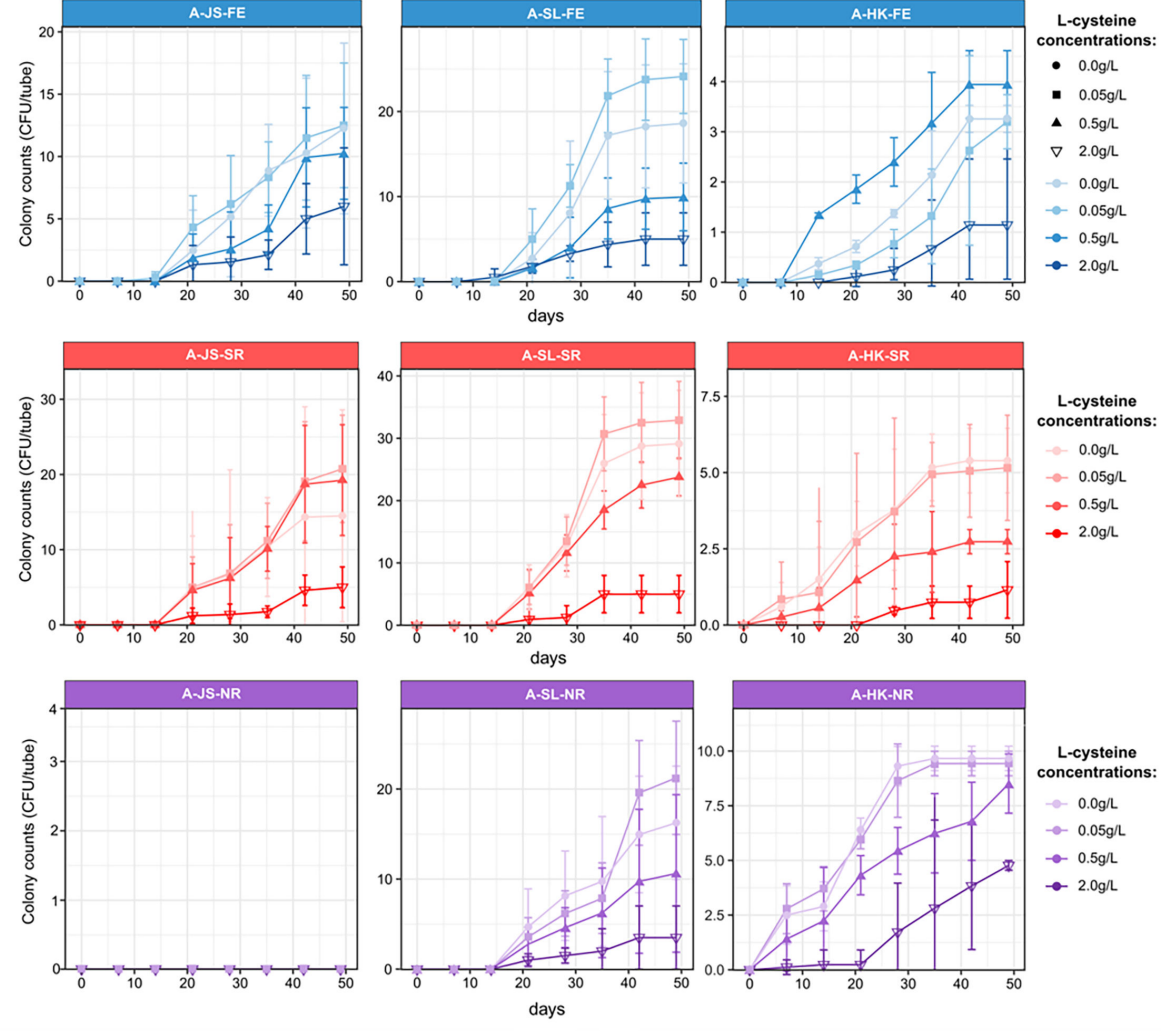
Total colony-forming units (CFU) in roll tubes using agar as the gelling agent. JS and SL represent samples from Jiangsu and Shengli oilfield, respectively, and HK represents the mangrove sediment sample in Hong Kong. The roll tubes were incubated under anoxic conditions including fermentative (FE), sulfate-reducing (SR), and nitrate-reducing (NR) conditions. CFU counts are averages from ten replicate roll tubes. Error bars represent standard deviations.

Generally, fewer colonies were formed in roll tubes using agar as the gelling agent across all cultivation conditions, indicating that there are fewer anaerobes capable of utilizing agar or its constituents compared with gellan gum in all samples (Fig. 2). Also, similar to the results of the gellan gum-made roll tubes, no colonies formed in the Jiangsu oilfield (group A-JS-NR), and HK samples showed relatively lower culturability under all three conditions.

In roll tubes based on either gelling agents, higher rates of increase in the colony were found in samples with 0.05 g L-Cys-HCl, followed by the no amendment samples, and then those amended with 0.5 g and 2.0 g L-Cys-HCl in the linear stage in several groups (G-JS-FE, G-JS-SR, G-SL-SR, G-SL-NR, A-JS-FE, A-SL-FE, A-SL-SR, A-SL-NR, and A-HK-NR). More colonies were also found in those without and with 0.05 g L-Cys-HCl samples when reaching the plateau. Although a longer lag phase was observed in the samples with no and 0.05 g L-Cys-HCl in group G-SL-FE, increased numbers of the colonies were formed after an extension of incubation time when compared with other samples with more L-Cys-HCl.

### Isolation of strains and determination of their phylogenetic properties

**Fig 3:**
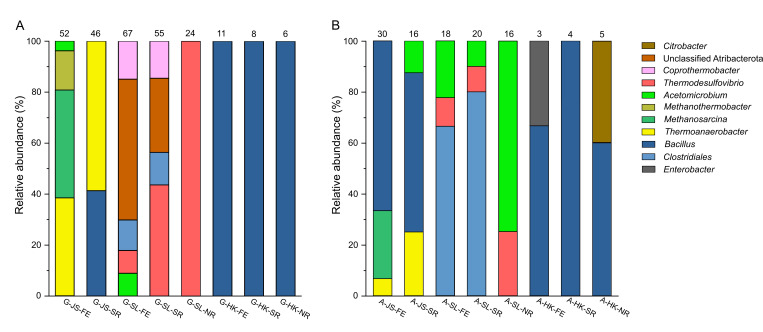
Phylogenetic distribution of isolates from roll tube using gellan gum (**A**) and agar (**B**) as the gelling agents. Isolates were classified at the genus level by SINA classifier based on SILVA (SSUr 138) database using their partial 16S rRNA gene sequences. The isolates from samples amended with different doses of L-Cys in each group were merged into one bar, and the number above each bar indicates the total number of isolates obtained from that group.

A total of 263 pure strains and 3 cocultures (containing 6 members in total) were isolated from samples containing gellan gum, and their partial 16S rRNA gene sequences were determined. The growth conditions and taxonomic classification of these isolates are summarized in Table S1. Since consistent lineage distributions were observed for samples amended with different doses of L-Cys within the same group (Table S1), we merged them into one column for each group (Fig. 3). All of the isolates recovered from the three HK groups (G-HK-FE, G-HK-SR, and G-HK-NR) were *Bacillus* species. The isolates from G-SL-FE and G-SL-SR were dominated by bacterial members from the genus *Thermodesulfovibrio* and unclassified Atribacterota and contained some *Coprothermobacter*, *Clostridium*, and *Lacrimispora*. However, isolates classified into *Acetomicrobium* were only isolated from G-SL-FE samples. The isolates recovered from the G-SL-NR group were all classified into *Thermodesulfovibrio*. As for JS groups, thermophiles of *Thermoanaerobacter* were found to be abundant in both G-JS-FE and G-JS-SR groups, whereas members of *Bacillus* were only found in the G-JS-SR group. *Acetomicrobium* and methanogens affiliated with *Methanosarcina* and *Methanothermobacter* were exclusively recovered from the G-JS-FE group. Growth occurred for most of the isolates affiliated with *Clostridium*, *Lacrimispora*, and unclassified Atribacterota after two transfers using the basal medium devoid of L-Cys, but containing 0.5% gellan gum, indicating the ability to utilize ingredients in gellan gum. Additionally, all the bacterial isolates and cocultures showed growth on glucose, a potential hydrolyzed monosaccharide from gellan (Table S1). Expectedly, except for the three *Methanosarcina* strains isolated as cocultures with a bacterial partner, no methanogens could grow solely on gellan or glucose.

As for the samples cultivated on agar, a total of 96 strains and 8 cocultures were retrieved (Table S2). The isolates from the three HK groups were mainly from *Bacillus*, and only one to two strains were found for the genera *Citrobacter* and *Enterobacter*. Isolates from A-JS-FE and A-JS-SR groups were mainly *Bacillus*, and to a lesser extent, strains of *Thermoanaerobacter*, *Acetomicrobium*, and cocultures of *Thermoanaerobacter* and *Methanosarcina* strains. In contrast, the isolates in A-SL-FE and A-SL-SR groups were mostly *Clostridium* species, followed by *Acetomicrobium* and *Thermodesulfovibrio*. The A-SL-NR group contained only *Acetomicrobium* and *Thermodesulfovibrio* but no *Clostridium*. Based on the growth test on agar, growth was confirmed for most strains from *Bacillus* and *Clostridium* after two transfers ( Table S2). While no growth was observed for strains of *Acetomicrobium*, *Citrobacter*, *Enterobacter*, *Thermoanaerobacter,* or *Thermodesulfovibrio* on agar, they can grow on galactose which is the major component of agar (6). Similar to the gellan gum experiment, no methanogen could grow solely on galactose.

Most of the isolated strains showed high similarity to each other based on the sequenced 16S rRNA gene sequences. A total of 18 representative strains were selected according to the OTUs clustered at 99% nucleotide identity of 16S rRNA gene sequence to explore their potential to utilize L-Cys (see Materials and Methods for details) (26). Among them, growth was confirmed in 10 strains representing species of *Acetomicrobium*, *Thermodesulfovibrio*, *Lacrimispora*, *Clostridium*, *Bacillus*, *Coprothermobacter*, *Citrobacter*, and *Enterobacter* after two transfers (Table S3). These phenotypes were largely consistent with the description of the previous studies (27–30).

**Fig 4:**
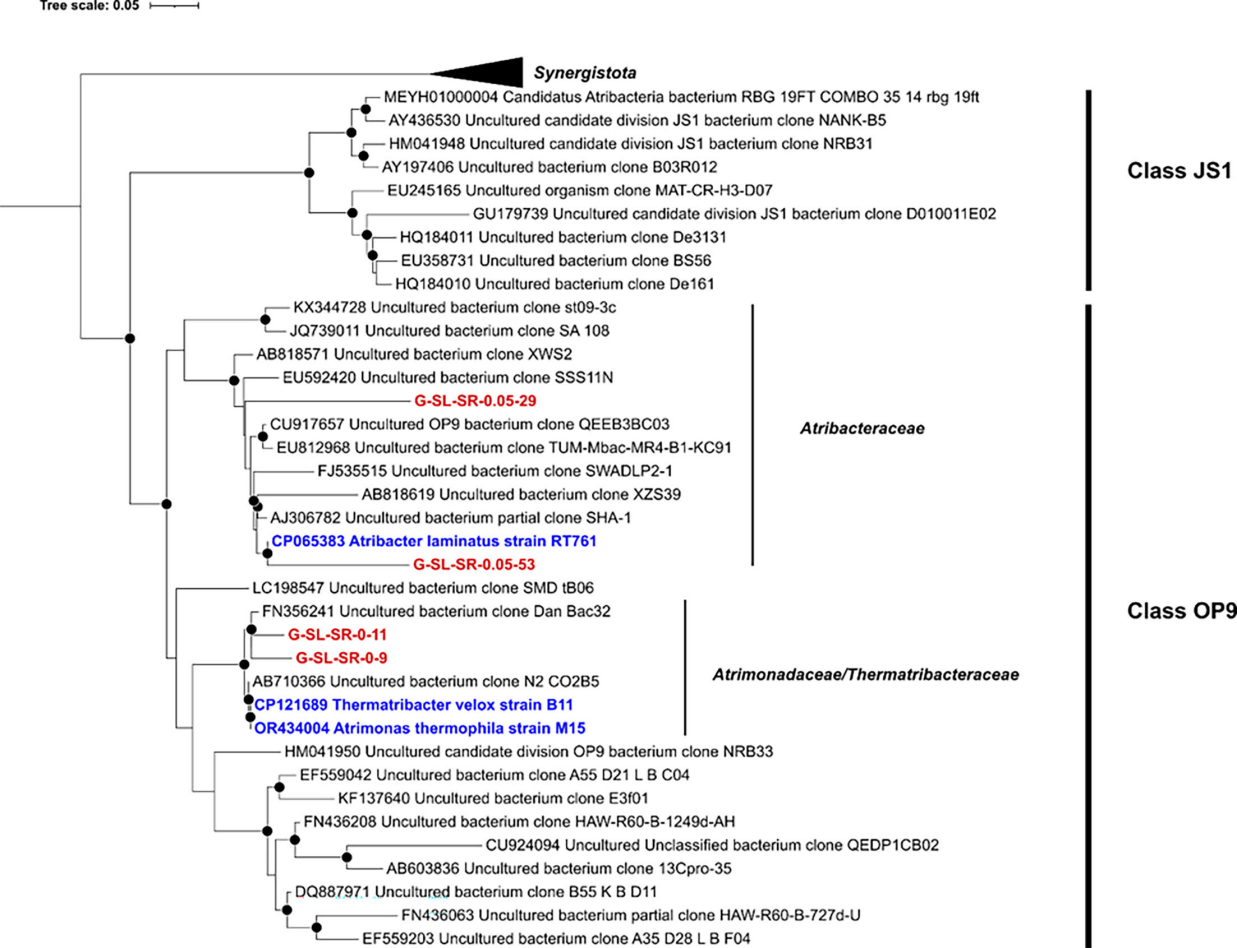
Phylogenetic positions of the novel atribacterial strains and their close relatives within the phylum Atribacteria. This maximum likelihood tree was built by the IQ-Tree method with the model TIM3+ F + I + G4 with 1,000 bootstrap replicates. Strains isolated in this study are in red, and the isolated reference strains are in blue. The scale bar indicates 0.10 substitutions per nucleotide position. Nodes with bootstrap values of >90%, >75%, and >50% are shown as black circles, gray circles, and open circles, respectively.

The partial 16S rRNA gene sequences from nearly all isolates mentioned above, except for those putative members of phylum Atribacterota, have more than 97% nucleotide sequence identities to the closest cultivars in the database, indicating that they are at least within the same genus of the corresponding cultivars. Therefore, we conducted further phylogenetic classification on four representative strains of Atribacterota which may represent novel taxa in the underrepresented phylum Atribacterota. Strains G-SL-SR-0.05-29 and G-SL-SR-0.05-53 were found to be closely related to the only cultured member within Atribacterota, *Atribacter laminatus* RT761 (96.6% and 97.1% identities, respectively). To obtain detailed phylogenetic information, we constructed a phylogenetic tree by using the atribacterial sequences publicly available (31). Consistent with the classification analysis, representative strains SL-SR-0.05-29 and SL-SR-0.05-53 were included in the family *Atribacteraceae* and clustered with *A. laminatus* RT761 (Fig. 4). However, strains G-SL-SR-0-11 and G-SL-SR-0-9 showed high 16S rRNA gene identities (96.4%–98.1%) to two recently isolated atribacterial strains, namely, *Thermatribacter velox* B11 and *Atrimonas thermophila* M15 (32, 33). In the phylogenetic tree, these two new strains together with the two previous isolates formed a cluster branched basally outside the family *Atribacteraceae* (Fig. 4). Based on the previous studies, atribacterial strains in this cluster may represent a novel family-level lineage, which has been tentatively named *Atrimonadaceae* or *Thermatribacteraceae* within the class OP9 (32, 33).

### Metabolic potential revealed by genomics

**Fig 5:**
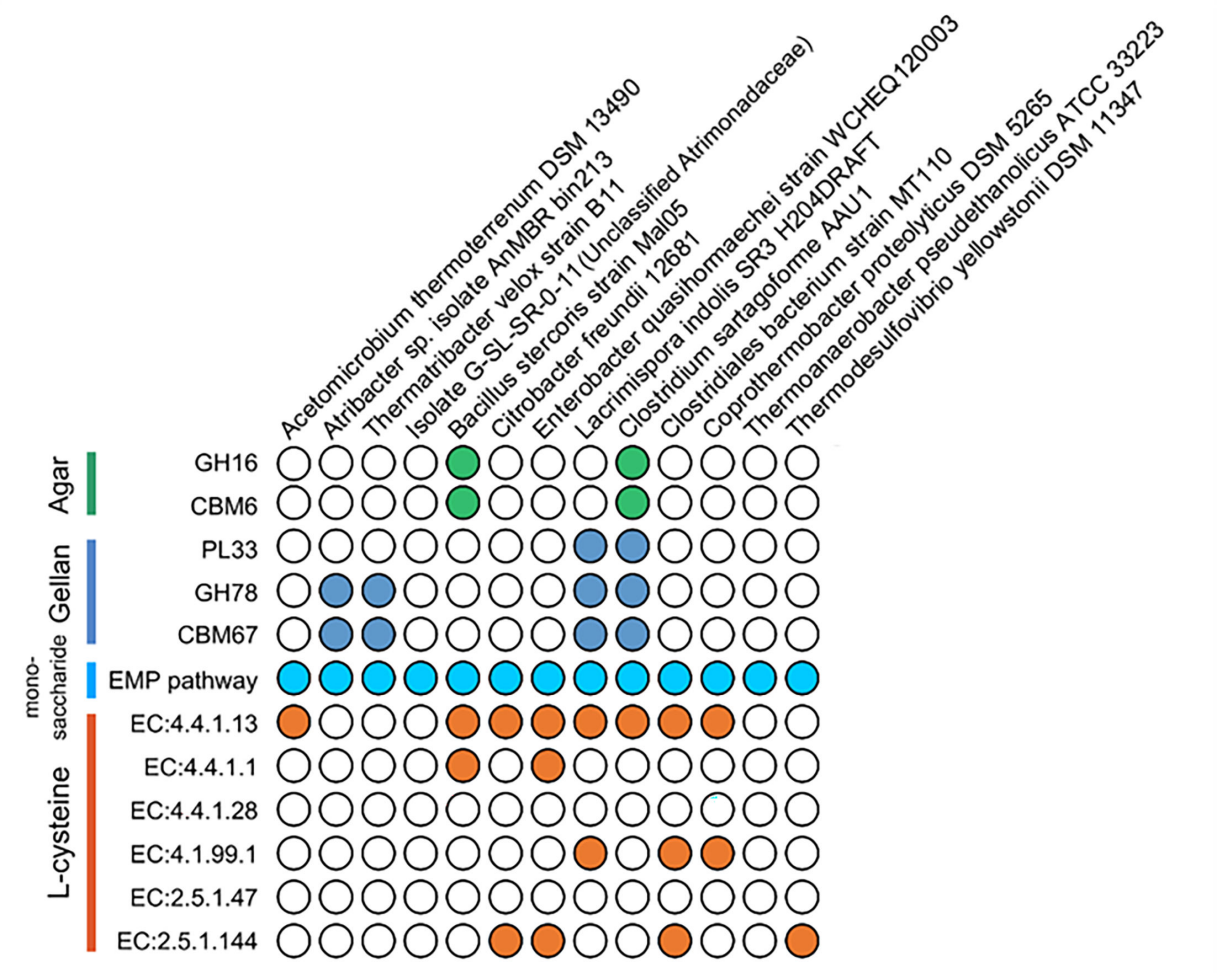
Occurrence of various genes in representative genomes and the sequenced genome of isolate G-SL-SR-0-11. GH16, *β*-agarase; PL33, endo-gellan lyase; GH78, *α*-L-Rhamnosidase. EC numbers: 4.4.1.13, cystathionine *β*-lyase (MetC and MalY); 4.4.1.1, cystathionine *γ*-lyase (CTH); 4.4.1.28, L-cysteine desulfidase (LCD); 4.1.99.1, tryptophanase (TNaA); 2.5.1.47, cysteine synthase (CysK); 2.5.1.144, Cys synthase B/O-acetylserine sulfhydrylase B (CysM). Closed circles indicated the presence of the specific gene.

To explore the metabolic potential of the isolates, their representative genomes (see Materials and Methods for details) and the newly sequenced genome of isolate G-SL-SR-0-11 (Table S4) were searched for genes responsible for agar, gellan gum, and L-Cys metabolism. Genes encoding endo-gellan lyase (PL33) were found in genomes representing *Lacrimispora* and *Clostridium* (Fig. 5), suggesting their ability to degrade gellan gum to tetrasaccharides (34). Additionally, genomes representing *Atribacter*, *Thermatribacter*, *Clostridium*, and *Lacrimispora* harbor genes encoding *α*-L-Rhamnosidase (GH78) which catalyzes the hydrolytic release of rhamnose from gellan gum and its carbohydrate-binding module (CBM67) (35). Genes encoding putative *β*-agarase (GH16) and its associated carbohydrate-binding module (CBM6) were only found in genomes representing *Bacillus* and *Clostridium*, indicating that these species may directly hydrolyze agar to oligosaccharides (3). Interestingly, all genomes contain genes that encode the enzymes of the complete Embden-Meyerhof pathway (Fig. 5), corroborating their growth on the monosaccharides, such as glucose and galactose. Consistent with the growth test on L-Cys, genes related to the desulfuration of L-Cys to pyruvate, including MetC, MalY, CTH, and CysM, were present in genomes representing members of *Acetomicrobium, Thermodesulfovibrio*, *Lacrimispora*, *Clostridium*, *Bacillus*, *Coprothermobacter*, *Citrobacter*, and *Enterobacter* (Fig. 5).

### Enrichment cultures

**Fig 6:**
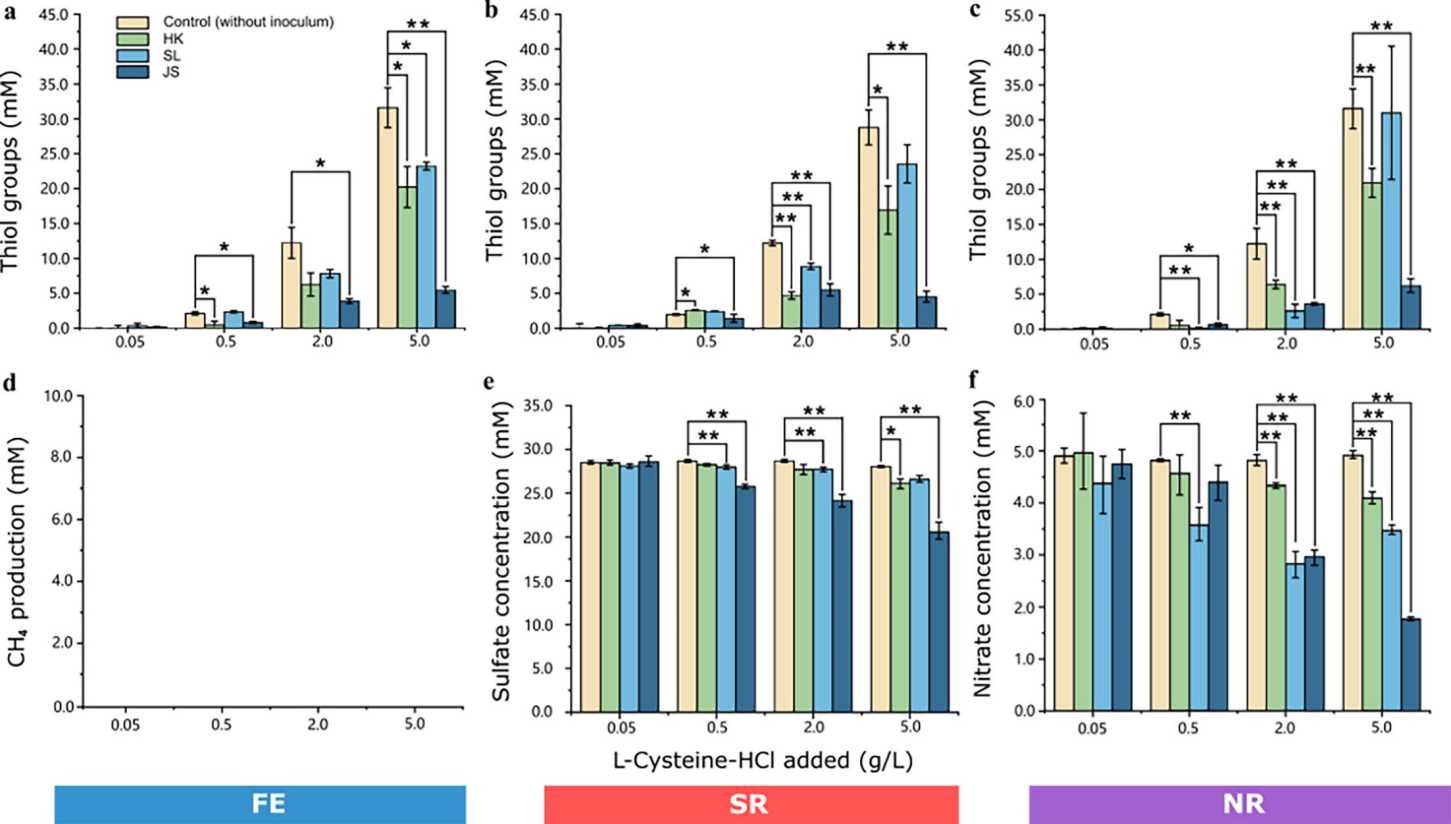
Utilization of L-Cys and electron acceptors and production of metabolites in cultures amended with different doses of L-Cys after cultivation. (**a–c**) The concentrations of free thiol groups in cultures after incubation under three conditions. (**d**) Detection of methane gas in the headspace of FE cultures; (**e**) concentration of sulfate in the medium of SR cultures; (**f**) concentration of nitrate in the medium of NR cultures. Error bars represent standard errors. Cultures with significant differences in L-Cys or electron acceptors (sulfate and nitrate) consumption when compared with those in the control samples were indicated by an asterisk (*P <0.01 or **P <0.001).

To avoid the effects of gelling agents as additional carbon and energy sources other than L-Cys, enrichment cultures devoid of gelling agents but containing L-Cys were set up to study the microbial community feeding on L-Cys. The enrichment cultures were established under the three anaerobic conditions at five different L-Cys concentrations (i.e., 0, 0.05, 0.5, 2.0, and 5.0 g/L). The concentrations of substrate L-Cys (represented by the free thiol group), electron acceptors, including SO_4_^2−^ and NO_3_^−^, as well as terminal products, such as CO_2_ and CH_4_, were measured after the incubation to determine the metabolic activities in the cultures (Fig. 6). To minimize the misinterpretation caused by the random error in measurement, only samples with significant differences (*P* <0.01 or *P* <0.001) in the concentrations when compared with those in the sterile control samples (without inoculum) were considered here. Overall, metabolic activities were found in some or all cultures amended with 0.5, 2.0, and 5.0 g L-Cys-HCl in all nine groups regardless of the different conditions and inocula. No obvious consumption of L-Cys was detected in all groups amended with no or 0.05 g L-Cys-HCl. Notably, no methane could be detected in all samples incubated under FE conditions, suggesting a lack of significant activity of methanogenesis in these cultures. Under SR conditions, the consumption of L-Cys showed an association with the decrease of sulfate in most of the samples, indicating a couple of L-Cys metabolism and sulfate reduction. Similarly, concurrent L-Cys consumption and nitrate reduction were also observed in several HK-NR and SL-NR samples, suggesting that L-Cys metabolism may be facilitated by NR microorganisms in these samples. However, no obvious nitrate reduction could be detected in the JS-NR group, even though L-Cys in these samples was largely consumed. This may suggest that the consumption of L-Cys in these samples is mainly performed by fermentation.

### Microbial compositions in the enrichment cultures

**Fig 7:**
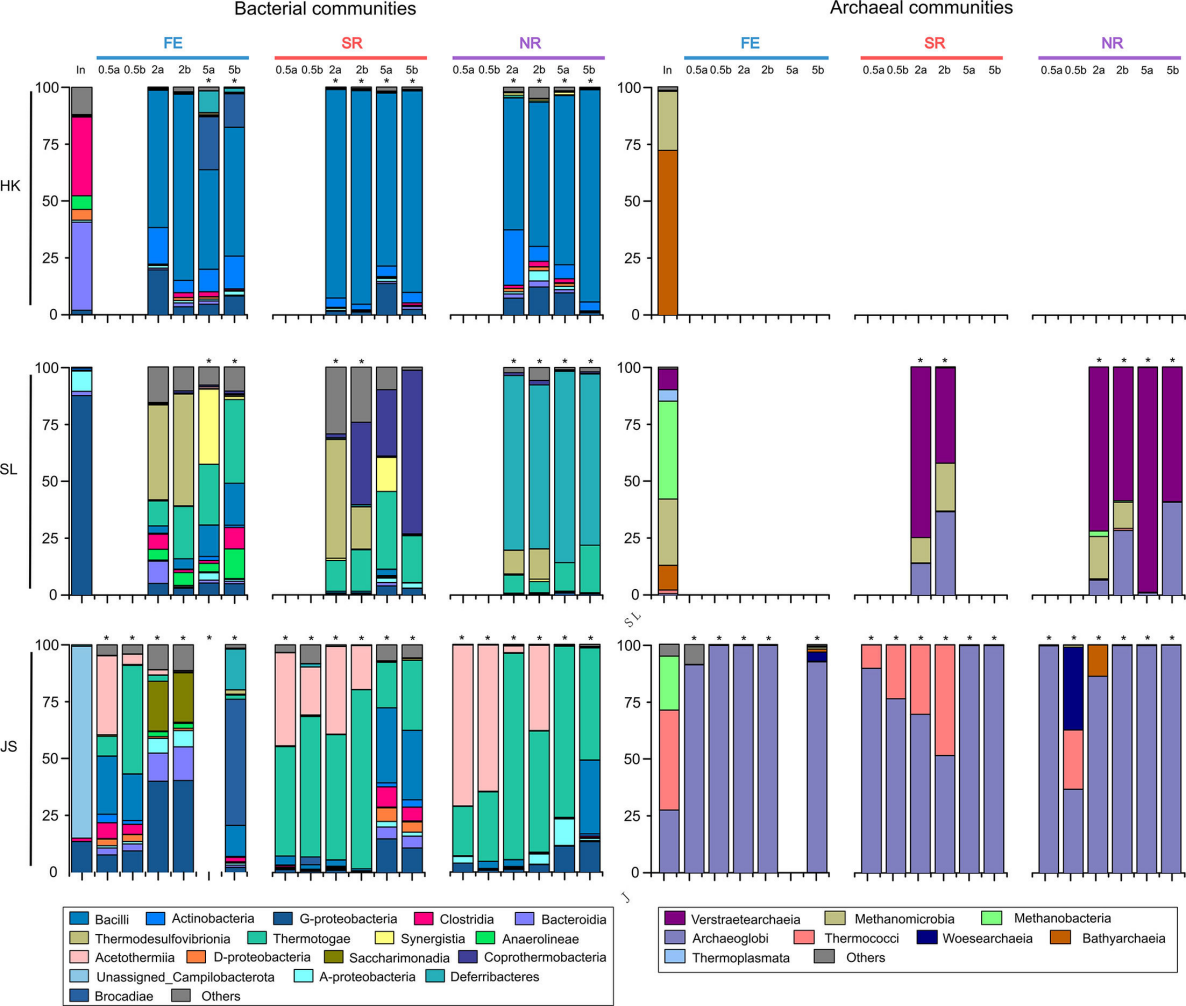
Microbial compositions at a class level based on 16S rRNA gene amplicons in three inoculum samples and cultures with 0.5, 2.0, and 5.0 g L-Cys-HCl amended. The samples with no and 0.05 g L-Cys-HCl which showed no significant microbial activity were not included here. The numbers above the columns represent the dose of L-Cys added, and the biological duplicates were indicated by letters “a” and “b,” and “In.” represents inoculum. Cultures without enough DNA materials for sequencing were not shown here. Cultures with significant differences (*P* <0.01) in Cys or electron acceptors (sulfate and nitrate) consumption, when compared with those in the control samples, were indicated by an asterisk.

Based on the metabolic analysis mentioned above, microbial biomass from the duplicates of enrichment cultures of all nine groups was harvested after incubation and investigated for microbial compositions. However, no sufficient DNA materials could be extracted from either of the no or 0.05-g-L-Cys-HCl cultures and some of the other cultures, probably due to the extremely low quantities of biomass (Fig. 7). This phenomenon demonstrated an association between the growth of microbial biomass with the concentration of L-Cys, suggesting that the growth of microbial communities mainly depends on L-Cys. 16S rRNA gene amplicon surveys revealed that the bacterial communities changed sharply after incubation when compared with those in the original samples, an indication of a microbial community shift after enrichment culturing. The HK cultures under all three conditions showed similar bacterial compositions, which were dominated by members from the class *Bacilli*, and *Actinobacteria* and *Gammaproteobacteria* were the minor ones. The dominance of *Bacilli* is consistent with the result of strain isolation from HK samples. On the contrary, the bacterial communities of SL groups were distinct under different incubation conditions. The bacterial communities of SL-FE-2 cultures were mainly composed of taxa of *Thermotogae*, *Thermodesulfovibrionia*, and *Bacilli*, whereas SL-FE-5 cultures mainly contained *Thermotogae* and *Bacilli* and some contained *Synergistia*. However, the SL-SR cultures were dominated by *Coprothermobacteria*, *Thermodesulfovibrionia*, and *Thermotogae*. The SL-NR cultures were found mainly composed of *Deferribacteres* and contained some *Thermodesulfovibrionia* and *Thermotogae*. The bacterial communities in JS-SR and JS-NR groups were similar, with *Acetothermiia* and *Thermotogae* being the major taxa in cultures amended with 0.5 and 2 g/L of substrates (JS-SR-0.5, JS-SR-2, JS-NR-0.5, and JS-NR-2). However, JS-SR-5 and JS-NR-5 cultures contained no members of *Acetothermiia*, whereas *Thermotogae*, *Bacilli*, and *Gammaproteobacteria* were found to be abundant in these cultures. Notably, the bacterial compositions in the JS-FE group varied greatly among cultures with different doses of the substrate. Taxa of *Acetothermiia*, *Thermotogae*, and *Bacilli* were major members in JS-FE-0.5 cultures, whereas *Gammaproteobacteria*, *Saccharimonadia*, *Bacteroidia*, and *Alphaproteobacteria* dominated the JS-FE-2 cultures. In JS-FE-5 cultures, *Bacilli*, *Brocadiae*, *Gammaproteobacteria*, and *Deferribacteres* were occasionally found to be abundant.

No archaeal members could be detected in all HK cultures under the conditions tested. As for SL cultures, archaeal members were only found in cultures of SL-SR-2, SL-NR-2, and SL-NR-5 conditions, which are all with obvious consumption of L-Cys and electron acceptors (Fig. 6). These archaeal communities are similar, which mainly consisted of taxa *Verstraetearchaeia*, *Methanomicrobia*, and *Archaeoglobi*. The archaeal communities in JS cultures are all dominated by *Archaeoglobi*, and *Thermococci* were found to be abundant in JS-SR cultures amended with 0.5 and 2.0 g/L substrate.

### Gene function profiles

Although we have observed the consumption of L-Cys in the enrichment cultures, the degradation pathway and the fate of the L-Cys were still unknown. Proteolytic bacterial members of the class Clostridia usually metabolize Cys through the Stickland reaction, oxidization to cystine, or desulfuration to pyruvate, ammonia, and sulfide (22). Fermentation of L-Cys via the Stickland reaction is characterized by the oxidation of one amino acid coupled with the reduction of another (36). However, amino acids such as glycine and proline which could serve as oxidants in the Stickland reaction were not added in the cultivation, and it is not likely that large quantities of such amino acids were introduced from the inocula (37). Therefore, the fermentation of Cys through the Stickland reaction may not be the main process in the enrichments. Alternatively, L-Cys could be oxidized to L-cystine. L-Cys may also be desulfured to pyruvate, ammonia, and sulfide, followed by pyruvate oxidation to acetate and CO_2_, and acetate could be further oxidized to CO_2_.

**Fig 8:**
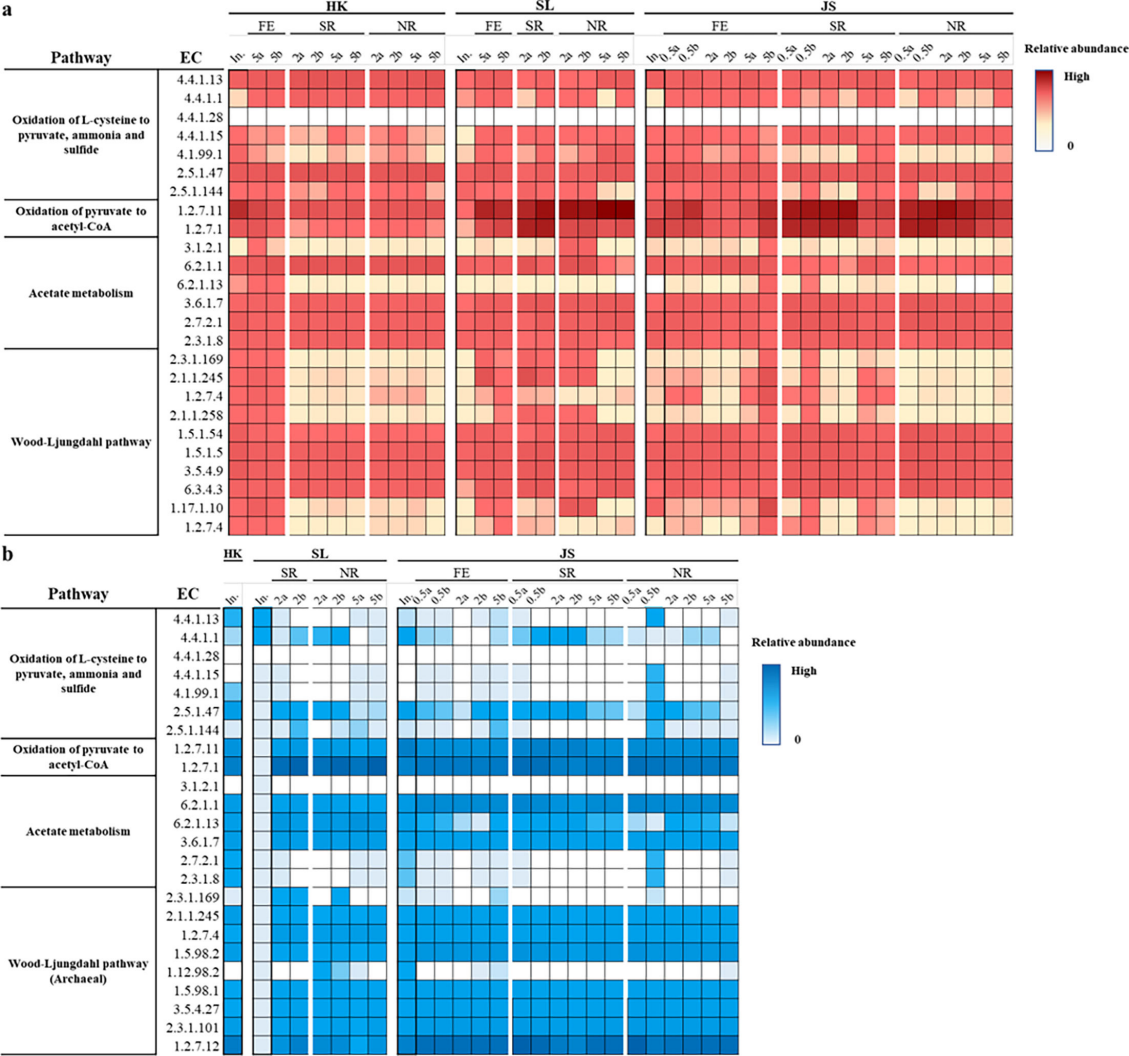
Predicted gene abundances in bacterial (**a**) and archaeal (**b**) communities in the cultures. EC numbers: 4.4.1.13, cystathionine *β*-lyase (MetC and MalY); 4.4.1.1, cystathionine γ-lyase (CTH); 4.4.1.28, L-cysteine desulfidase (LCD); 4.4.1.15, D-cysteine desulfhydrase; 4.1.99.1, tryptophanase (TNaA); 2.5.1.47, cysteine synthase (CysK); 2.5.1.144, Cys synthase B/O-acetylserine sulfhydrylase B (CysM); 1.2.7.11, 2-oxoglutarate/2-oxoacid ferredoxin oxidoreductase; 1.2.7.1, pyruvate ferredoxin oxidoreductase; 3.1.2.1, acetyl-CoA hydrolase; 6.2.1.1, acetyl-CoA synthetase; 6.2.1.13, acetate-CoA ligase (ADP-forming); 3.6.1.7, acylphosphatase; 2.7.2.1, acetate kinase; 2.3.1.8, phosphate acetyltransferase; 2.3.1.169, acetyl-CoA decarbonylase/synthase, CODH/ACS complex; 2.1.1.245, acetyl-CoA decarbonylase/synthase, CODH/ACS complex; 1.2.7.4, anaerobic carbon-monoxide dehydrogenase, CODH/ACS complex; 2.1.1.258, 5-methyltetrahydrofolate corrinoid/iron sulfur protein methyltransferase; 1.5.1.54, methylenetetrahydrofolate reductase (NADH); 1.5.1.5, methylenetetrahydrofolate reductase (NADH); 3.5.4.9, methylenetetrahydrofolate dehydrogenase (NADP+)/methenyltetrahydrofolate cyclohydrolase; 6.3.4.3, methylenetetrahydrofolate dehydrogenase (NADP+)/methenyltetrahydrofolate cyclohydrolase/formyltetrahydrofolate synthetase; 1.17.1.10, formate dehydrogenase (NADP+); 1.5.98.2, 5,10-methylenetetrahydromethanopterin reductase; 1.12.98.2, 5,10-methenyltetrahydromethanopterin hydrogenase; 1.5.98.1, methylenetetrahydromethanopterin dehydrogenase; 3.5.4.27, methenyltetrahydromethanopterin cyclohydrolase; 2.3.1.101, formylmethanofuran--tetrahydromethanopterin N-formyltransferase; 1.2.7.12, formylmethanofuran dehydrogenase. Relative abundance values are log-transformed. The numbers above the columns represent the dose of L-cysteine added, and the biological duplicates were indicated by letters “a” and “b,” and “In.” represents inoculum. Only experimental groups with significant differences (*P* <0.01) in L-cysteine or electron acceptor (sulfate and nitrate) consumption when compared with those in the control samples were shown here.

PICRUSt analysis was carried out to predict the putative metabolic properties of microbial communities in these samples. For better insight, we have split the taxonomic assignment data into archaeal and bacterial reads separately (Fig. 8).

Genes encoding the cystine reductase for oxidation of L-Cys to L-cystine were not recovered in all samples, probably due to the underrepresentation of such genes in the KEGG database. Most of the genes encoding L-Cys desulfurase were found in abundance in bacterial communities rather than in the archaeal communities. Although different types of L-Cys desulfurase genes were found enriched among bacterial communities of HK, JS, and SL samples, the fact that they all contained plentiful‌ L-Cys desulfurase genes in all groups indicated the potential of L-Cys desulfuration. Pyruvate could be converted to acetyl-CoA, followed by acetate formation. Genes for 2-oxoacid oxidoreductase (ferredoxin) and pyruvate:ferredoxin oxidoreductase were more abundant in the bacterial communities of JS and SL samples than in HK samples, whereas the archaeal communities from all samples contained a considerable abundance of pyruvate:ferredoxin oxidoreductase genes. The ability of acetate generation was well-conserved in both bacterial and archaeal communities in all samples, supported by the generally high levels of genes related to acetate metabolism. In bacterial anaerobes, acetate could be further oxidized to CO_2_ through the Wood-Ljungdahl pathway, and a counterpart pathway also exists in archaeal anaerobes which share multiple genes with hydrogenotrophic methanogenesis (38, 39). Most of the genes associated with the bacterial Wood-Ljungdahl pathway were found enriched in SL cultures compared with HK and JS samples. Although genes in the archaeal type Wood-Ljungdahl pathway were found to be abundant in all samples, they were mainly encoded in methanogens which may function in methane production from CO_2_, rather than the reversal of acetate oxidation to CO_2_.

## DISCUSSION

### Isolated strains utilizing gellan gum and agar

The observed improvement in culturability of samples amended with no or 0.05 g L-Cys-HCl compared with those samples with 2.0 g L-Cys-HCl was probably caused by the abiotic reaction of sulfide and the residual oxygen (40). In samples amended with 0.5 g and 2.0 g L-Cys-HCl where enough L-Cys was added to scavenge the trace of oxygen in the medium, a colorless medium without oxygen was achieved before adding the Na_2_S stock solution. However, a medium with a slightly pink color was observed for no and 0.05 g L-Cys-HCl due to the high redox potential caused by the residual oxygen from medium preparation. The remaining oxygen in those media may react with Na_2_S to form elemental sulfur and therefore reduce the concentration of sulfide in the medium. It has been reported that even low sulfide concentrations could be toxic and inhibitory to microorganisms from freshwater or brackish habitats (40). Additionally, the elemental sulfur may provide additional electron acceptors for microorganisms capable of sulfur reduction. Hence, the samples with no and 0.05 g L-Cys-HCl may benefit from the combined effects of decreased concentrations of sulfide and the formation of elemental sulfur. Furthermore, the slight enhancement of culturability of most FE and SR samples with 0.05 g L-Cys-HCl compared with the control samples may result from the low concentration of L-Cys, which may both serve as electron carriers during interspecies electron transfer and as supplies of L-Cys for L-Cys auxotrophy of microorganisms (41).

Based on the combined evidence of the growth test and the presence of genes responsible for gellan lysis, *Atribacter*, *Clostridium*, *Lacrimispora*, and *Thermatribacter* are reported to be anaerobic gellan gum degraders for the first time. However, despite the success in the growth of isolate G-SL-SR-0-11 on gellan gum, no genes associated with gellan degradation could be found in its genome. Therefore, these atribacterial strains represented by isolate G-SL-SR-0-11 may utilize a hitherto uncharacterized gellan lyase to degrade gellan. It also cannot be completely ruled out that they may utilize the hydrolyzed monosaccharides (for instance, glucose), α-hydroxy ketone, or α-hydroxy aldehyde contaminated in the gellan gum for growth (28, 42, 43). This hypothesis is supported by their growth on glucose and the presence of a complete set of genes responsible for the EMP pathway in their representative genome.

As for anaerobic agar degradation, genes for agar degradation were found in the representative genome of *Bacillus* species, corroborating the previous work (44). Moreover, this is the first report that some isolates affiliated with the genus *Clostridium* were able to grow on agar as the substrate, and genes for agar degradation were also found in their representative genome. Although the taxonomic distribution of agarolytic strains isolated here is distinct from the previous studies, this result is reasonable since previous isolations were mainly conducted under aerobic conditions (4).

Based on the growth test, non-gel degraders from *Acetomicrobium*, *Coprothermobacter*, *Citrobacter*, *Enterobacter*, and *Thermodesulfovibrio* were predicted to mainly utilize L-Cys added in the roll tube. The formation of colonies of *Thermoanaerobacter*, as well as its cocultures with methanogens, on roll tubes, is probably facilitated by utilizing other saccharide compositions hydrolyzed from agar by extracellular lyase secreted from those agar degraders or generated during medium preparation (6).

Most of the isolates recovered from HK samples were assigned to a single genus of *Bacillus* regardless of the type of available electron acceptor. Members of *Bacillus* are capable of both fermentation and nitrate reduction, which may confer their competitive advantages under different incubation conditions (45). The lowest number of the colony was formed in HK samples, which is probably due to the extremely low abundance of *Bacillus* in the inoculum of HK mangrove sediments (Fig. 8). In SL samples, strains of *Thermodesulfovibrio* were predicted to respire sulfate in SR condition and ferment in FE condition based on the previous physiological study (46). Nevertheless, members of *Atribacterota*, *Acetomicrobium*, *Coprothermobacter*, and *Lacrimispora* probably perform a fermentative lifestyle in SL samples (29, 31). Although incapable of nitrate reduction, isolates of *Thermodesulfovibrio* and *Acetomicrobium* were recovered from SL-NR samples, and they are predicted to perform fermentation (42, 46). The closest relatives of *Thermoanaerobacter* strains recovered from JS-FE and JS-SR samples have been described as fermentative thermophilic strains and were also isolated from the oilfield ecosystem (47). Therefore, these strains may perform fermentation under those two conditions. Methanogens affiliated with genera *Methanosarcina* and *Methanothermobacter* were exclusively isolated from JS samples incubated without exogenous electron acceptors (FE condition). These methanogens were predicted to utilize the hydrogen, CO_2_, and acetate produced by the partner bacteria during gel degradation (48).

Previous studies have reported that the use of gellan gum as a gelling agent might lead to the cultivation of yet-to-be-isolated organisms and might also increase the opportunity to study slow-growing microorganisms in a wide variety of ecosystems (8, 14). In this study, we isolated four strains representing novel taxa in the phylum Atribacterota which, so far, contains only three cultivars from SL samples (31). These strains were affiliated with two different family-level lineages of Atribacterota in the phylogenetic tree (Fig. 4), indicating a high diversity of Atribacterota in the oilfield ecosystem. These results and further study of these novel members may greatly expand the diversity and metabolic capabilities of the newly discovered members of Atribacterota.

It also indicates that the mesophilic microbial communities in the mangrove sample contain rare and low diversity of anaerobic gellan- and agar-degrading microorganisms. In contrast, it is much enhanced in the thermophilic microbial communities from oil production water samples in terms of colony formation and the diversity of isolates. However, a comprehensive study requiring more diverse samples from ecosystems with different temperatures is necessary to give a systematic comparison of the abundance and diversity of gellan- and agar-degrading microorganisms between mesophilic and thermophilic communities.

### Microbial communities feeding on L-cysteine

According to the metabolite analysis, inocula of the production water from SL oilfield and sediment sample from HK mangrove contained fermentative, sulfate-reducing and nitrate-reducing microbial communities which metabolize L-Cys, whereas the inoculum from JS oilfield only contained fermentative and sulfate-reducing microbial communities metabolizing L-Cys. Especially, we observed significant L-Cys and electron acceptors (sulfate and nitrate) consumption and microbial community shift in groups amended with 0.5 g/L of L-Cys-HCl, which is the regular amount added in laboratory media. This result demonstrates that the routine use of L-Cys as a reducing agent in anaerobic culturing and experiments must be re-examined, as it can greatly affect and even falsify the interpretation of experiments.

The dominant bacterial members of the genus *Bacillus* in HK samples have been reported to metabolize L-Cys using L-Cys desulfurase (49), and the isolates of *Bacillus* species were also proved to grow on L-Cys as mentioned above. In SL samples, the bacterial members of *Thermotogae* across all groups also contained L-Cys desulfurase, indicating a potential to ferment L-Cys (50). Members of *Thermodesulfovibrionia* in all SL groups may utilize L-Cys as shown above (46). The enriched *Coprothermobacteria* in SL-SR and communities may metabolize L-Cys by fermentation (29), which has been verified by the isolated *Coprothermobacter* strains in this study. *Deferribacteres* in SL-NR communities may couple L-Cys metabolism to nitrate reduction (51). Apart from *Thermotogae* mentioned above, members of an uncultured phylum, *Acetothermia* (Bipolaricaulota, former OP1), were found to be abundant in bacterial communities of JS-SR and JS-NR samples. Previous *in silico* analysis has predicted the metabolic capabilities of homoacetogenic fermentation of sugars and amino acids in all lineages of *Acetothermia* (52, 53). Consistent with the little nitrate reduction in the medium, no known NR microorganisms were enriched in JS-NR samples.

As for archaeal communities, the failure to detect archaeal reads by 16S rRNA gene amplicon sequencing in all HK samples and SL-FE samples suggests the extremely low abundance of archaeal members in these cultures. Although methanogens of *Methanomicrobia* were found in archaeal communities of SL-SR and SL-NR samples, no methane could be detected after the incubation. This could be explained by their relatively low abundance in the communities and low activities under the relatively high redox potentials of SR and NR conditions. The dominant *Verstraetearchaeota* in SL archaeal communities under all conditions was an underrepresented archaeal phylum. The recent isolation and cultivation of members of *Verstraetearchaeota* showed that they are strict hydrogen-dependent methylotrophic methanogens with methanol and monomethylamine as electron acceptors and hydrogen as electron donors (54, 55). Nevertheless, a metatranscriptomic analysis is required to delineate the transcriptional activities of these methanogens in the cultures. Based on the previous results that *Archaeoglobi* cannot grow on L-Cys, members of *Archaeoglobi* and probably *Thermococci* as well were predicted to utilize the metabolites generated by bacterial members in the JS samples (56). More specifically, the growth of *Thermococcus* could be facilitated by reducing elemental sulfur (S^0^) resulting from oxygen contamination in the medium (57), whereas *Archaeoglobi* may utilize sulfate as electron acceptors in JS-SR and SL-SR samples (42).

It is noteworthy that the isolated strains capable of metabolizing L-Cys are generally present in these microbial communities. It is also expected that more diverse microorganisms from the communities were found than those isolates since many microorganisms may not be culturable in the roll tubes, or some of them may feed on metabolites generated by other partners rather than L-Cys (27).

The potential to metabolize L-Cys in the microbial communities was also supported by the predicted presence of L-Cys desulfurase genes in the samples. However, genes responsible for L-Cys oxidation to L-cystine were underestimated here due to a lack of representative genes deposited in databases (such as NCBI and KEGG databases). To overcome this shortage and to give a comprehensive description of the metabolism pathway of L-Cys in microbial communities, metagenomic sequencing analysis is warranted in the future.

In the post-metagenomics era, precise information about the physiological, taxonomy, and metabolism of microorganisms is gaining more and more attention to validate the increasing number of predictions based solely on non-cultured genome information (1). Cultivation in the laboratory is no doubt the most valuable strategy available for obtaining that information (58). However, it has been widely accepted that the number of viable microorganisms in laboratory media is several orders of magnitude lower than the number of microorganisms in the environment, which is called a “great plate count anomaly” (59, 60). There are several reasons accounting for this number gap, such as the slow-growing microorganisms would be neglected in the regular incubation time, and the nutrient-rich laboratory media exert a selective effect on isolating fast-growing copiotrophs, hence wiping out slow-growing oligotrophs. In this study, we proposed another explanation for this phenomenon, that is, the common ingredients, gelling agent (agar and gellan gum), and reducing agents (L-Cys-HCl and probably titanium (III) citrate as well) in the laboratory media may provide extra carbon and energy sources to microorganisms and therefore decrease the isolation effectiveness and specificity of such experiments where the supplemented substrates are supposed to be the only carbon and energy source. Although the ecosystem types of the inoculum in this study are limited to oil reservoirs and mangrove sediment, the isolated and enriched microorganisms are cosmopolitan taxa that are widely distributed, and usually abundant, in different biotypes. Therefore, these overlooked carbon and energy sources may have a common effect on the culturing experiments of anaerobic microorganisms from different environments. In this case, inorganic reducing agents such as sulfide sodium and amorphous ferrous iron could be used in place of L-Cys-HCl and titanium (III) citrate that contain organic carbon (20, 31, 61). As for strain isolation, methods like dilution-to-extinction without the addition of gelling agents are suggested for enumeration purposes (62).

## MATERIALS AND METHODS

### Sample collection

The two oil production water samples were collected from Jiangsu oilfield (Well W2-71, 66°C), China, on 17 May 2020 and Shengli oilfield (Block Z3, 52°C), China, on 2 September 2020, respectively. Its physicochemical characteristics have been described elsewhere (39, 63). The mangrove soil sample was taken from Mai Po Nature Reserve in Hong Kong, China, on 23 December 2010 (21).

### Preparation of media

The Hungate roll-tube medium used for the isolation of microorganisms from these environmental samples was prepared as previously described (13). The basal mineral medium (per liter distilled water) contained 2.85 g of NaCl, 0.15 g of MgCl_2_·6H_2_O, 0.22 g of CaCl_2_·2H_2_O, 0.25 g of NH_4_Cl, 0.09 g of MgSO_4_, 1.49 g of KCl, and 1 mg resazurin, and 1.0 mL of multiple vitamin stock solution, 1.0 mL of trace mineral element stock solution, and 5% Na_2_S stock solution (final concentration of 2.56 mM) were added after autoclaving. To test the effect of different gelling agents, one of the three gelling agents including gellan gum (2%, Yuanye, Shanghai, China; Sigma-Aldrich, Shanghai, China) and agar (3%, Sigma-Aldrich, Shanghai, China) was added to make solid medium. Different amounts of L-Cys-HCl were added to the media. Based on the fact that 0.5 g/L of L-Cys-HCl is usually the amount used in anaerobic cultivation, the media were amended with 0.05 g, 0.5 g, or 2 g of L-Cys-HCl. Either 4.0 g of Na_2_SO_4_ or 0.43 g of NaNO_3_ were added into the respective medium as electron acceptors for preparation of sulfate-reducing and nitrate-reducing cultivation conditions, respectively, whereas medium without any extra electron acceptors was made as the fermentative condition (64). An inoculum of 1.5% of the production water or suspension from the sediment sample was used for roll-tube cultivation. The basal medium was boiled and cooled under a stream of pure N_2_ gas, and aliquots of 6 mL were dispensed into each Hungate tube with ten replicates. Media used for enrichment experiments contained the same ingredients as those of the Hungate roll tube except for gellan gum with biological duplicates. An inoculum of 3% of production water or sediment suspension from the mangrove wetland sample was used for enrichment cultivation.

The single clone was picked after reaching the plateau period of total colony number, cultured in a basic liquid mineral medium amended with the corresponding substrates (0.5 g/L of one of the three gelling agents or 20 mM L-Cys) and electron acceptors as mentioned above. After at least two transfers (3% inoculum), the growth of cells was checked by a phase contrast microscope (OLYMPUS-CX43), and DNA was extracted from the culture medium to confirm the growth and purity of the culture.

### Phylogenetic analysis of isolates

DNA extraction of the isolates was conducted as previously described (65). Two pairs of universal primers of domains Archaea and Bacteria were used for PCR amplification of 16S rRNA gene: Arc109F (5′-ACKGCTCAGTAACACGT-3′) and Arc915R (5′-GTGCTCCCCCGCCAATTCCT-3′) or bac27F (5′-AGAGTTTGATCMTGGCTCAG-3′) and bac915R (5′-GGTTACCTTGTTACGACTT-3′). Phylogenetic classification of each amplicon was performed using the SINA classifier based on SILVA (SSUr 138) database (66, 67). The closest relatives of each OTU were inferred by using the BLAST program against NCBI ‘*nr-nt*’ database (68). Almost-full-length 16S rRNA gene sequences were determined by direct sequencing of the DNA fragment amplified with the primer pair of 27F and 1492R (5′-GGTTACCTTGTTACGACTT-3′), as described previously (13). Phylogenetic analysis was performed as described previously (63). In brief, 16S rRNA gene sequences were aligned using MAFFT with parameters ‘--adjustdirectionaccurately --maxiterate 1000 --globalpair --anysymbol’ (69). Alignments were then checked, and columns containing >95% gaps were trimmed in aliview (43). Maximum-likelihood trees were constructed in the IQ-Tree method v1.6.7 with ‘-m TEST -bb 1000 -alrt 1000’ and viewed in MEGA7 (70, 71).

### Chemical analyses

Methane, hydrogen, and carbon dioxide in the headspace of cultures were periodically monitored using gas chromatography (GC, Agilent 6890) equipped with a stainless-steel column filled (1.5 m; 5 Å carbon molecular sieves) and a flame ionization detector and a thermal conductivity detector (65). The net production of methane and carbon dioxide was calculated after subtracting the detected amount of those in substrate-free controls. SO_4_^2−^ was quantified by ion chromatography (ICS-1100, U.S.) which was equipped with an IONPAC AS11-HC column (temperature, 30℃; eluent, KOH gradient elution; detector, conductivity detector and ampere detector; suppressor, anion suppressor) and an IONPAC CS12A column (temperature, room temperature; eluent, 20 mM methylsulfonic acid isocratic elution; detector, conductivity detector; suppressor, cation suppressor).

The concentration of NO_3_^−^ in the medium was measured by reacting with 2, 4-phenoldisulfonic acid (72), which was produced by sulfonation and decarboxylation of salicylic acid and concentrated sulfuric acid. Then, 6-nitro-2, 4-phenoldisulfonic acid was produced and formed a yellow solution after molecular rearrangement under alkaline conditions. The absorbance at 410 nm against a control without nitric acid was measured using a spectrophotometer.

The free thiol group was measured using the total thiol group kit (Hefei Lyle Biotechnology Co., Ltd.). The main principle is free thiols and 5, 5-dithiobis (2-nitrobenzoic acid) (DTNB) reaction, under neutral or basic conditions to form a yellow compound, which has a maximum absorption peak at 412 nm wavelength (41). The absorbance value was read by a microplate reader. Before formal determination, samples with large concentration differences were selected for prediction. When the absorbance value was greater than 1, the medium solution was diluted before measurement.

### Retrievement of representative genomes and genome sequencing

The representative genome of each representative strain was retrieved by searching for the genomes which contain the most similar 16S rRNA gene sequences to the OTU sequences using the BLAST program against NCBI ‘*refseq_genomes*’ and ‘*wgs*’ database. Except for the two OTUs representing atribacterial members of the newly defined family of Atrimonadaceae/Thermatribacteraceae, most representative genomes showed high 16S rRNA gene identities (more than 99%) to the OTU sequences, suggesting a close relation between the representative strains and the genomes (Table S4). Then, the total genomic DNA of strain G-SL-SR-0-11 was extracted for genome sequencing as mentioned previously (13). The detailed sequencing procedures and gene annotation can be found in Supplementary Materials.

### Microbial community analysis

The DNA extracted from enrichments was subjected to 16S rRNA gene amplicon sequencing on an Illumina Hiseq 2500 platform, and 250 bp paired-end reads were generated. As previously described, the V3−V4 region of the bacterial 16S rRNA gene was amplified using the primer pairs 338F and 806R/515F, and the V4−V5 region of the archaeal 16S rRNA gene was amplified using the 806R/515F and 907R (73). The valid sequences were clustered into OTUs at a 99% species cutoff and classified against SILVA release 138 sequences.

### Function prediction and metabolic pathway reconstruction

PICRUSt2 was used to predict the functional composition of each community metagenome (74).

Command ‘place_seqs.py’ was used to insert the representative sequences generated from the previous microbial composition analysis into a reference tree which is based on 20,000 16S sequences from genomes in the Integrated Microbial Genomes database. The script ‘hsp.py’ was used to predict copy number of gene families and the copy number of 16S rRNA gene sequences for each OTU. Then, BIOM tables of the abundance of each OTU and gene predicted in the last step were used as input files using ‘metagenome_pipeline.py’ command to predict EC number abundances in all samples. Any OTUs nearest-sequenced taxon index >2 will be excluded from the following analysis, and the read depth per OTU is divided by the predicted 16S copy numbers.

## Data Availability

The nucleotide sequence data for the partial 16S rRNA genes of isolated OTUs are deposited in GenBank under accession numbers OP869989-OP870007 and PQ866049-PQ866051, and the high-throughput 16S rRNA gene sequence data and the complete genome sequence data of strain G-SL-SR-0-11 have been submitted to GenBank under Bioproject PRJNA902943.

## AFTER EPUB

*[This article was published on 12 February 2025 with errors in Fig. 1. The figure was corrected in the current version, posted on 18 February 2025.]*

## SUPPLEMENTAL MATERIAL

Supplemental material aem.02276-24-s0001.docx Tables S1 to S4; Figure S1.

ASM does not own the copyrights to Supplemental Material that may be linked to, or accessed through, an article. The authors have granted ASM a non-exclusive, world-wide license to publish the Supplemental Material files. Please contact the corresponding author directly for reuse.
