# Supplementary material for: Gelling and reducing agents are potential carbon and energy sources in culturing of anaerobic microorganisms
Source: Appl Environ Microbiol. 2025 Feb 12;91(3):e02276-24. doi: 10.1128/aem.02276-24 (PMC11921371; doi:10.1128/aem.02276-24)
Supplement: Supplemental material — Tables S1 to S4; Figure S1. [file aem.02276-24-s0001.docx]

**Supporting Information**

**Gelling and reducing agents are potential carbon and energy sources in culturing of anaerobic microorganisms**

Yi-Fan Liu^1,2,#^, Liu Yang^1,2,#^, Qing-Ping He^1,2^, Yi-Lin Xu^1^, Yu-Tong Zhu^1^, Yan-Le Mi^1^, Lei Zhou^1,2^, Shi-Zhong Yang^1,2^, Ji-Dong Gu^3^ and Bo-Zhong Mu^1,2,^*

^1^ State Key Laboratory of Bioreactor Engineering and School of Chemistry and Molecular Engineering, East China University of Science and Technology, 130 Meilong Road, Shanghai 200237, P.R. China

^2^ Engineering Research Center of MEOR, East China University of Science and Technology, 130 Meilong Road, Shanghai 200237, P. R. China

^3^ Environmental Science and Engineering Group, Guangdong Technion Israel Institute of Technology, 241 Daxue Road, Shantou, Guangdong 515063, P.R. China

* Correspondence: Bo-Zhong Mu, State Key Laboratory of Bioreactor Engineering and School of Chemistry and Molecular Engineering, East China University of Science and Technology, Shanghai 200237, 130 Meilong Road, Xuhui District, P.R. China.

E-mail: bzmu@ecust.edu.cn

**The file contains:**

Pages: 27

Supplementary Table: 4

Supplementary Figure: 1

**Supplementary Methods and Materials**

*Genome Sequencing and gene annotation*

The total genomic DNA of strain L-13 was extracted using the Wizard Genomic DNA purification kit by following the manufacturer’s instructions. DNA quantity and quality were determined with a Nanodrop ND-100 UV–Vis Spectrophotometer and a Qubit 2.0 fluorometer. Genome sequencing was performed using both an Illumina Hiseq×10 platform and a PacBio RS II platform at Majorbio Company (Shanghai, China). For Illumina libraries, the insert size was 400 bp with a pair-end sequencing length of 150 bp. After filtering and cutting the adapters, sequence assembly was carried out using SPAdes .(1)

The open reading frames (ORFs) of the scaffolds were predicted by Prodigal (v2.6) with “meta” option. The read mapping files were used to calculate the index of replication (iRep) of MAGs. Annotation of the ORFs was performed using several databases: (2)v5.0, GhostKOALA(3) and KofamKOALA(4). Additionally, The carbohydrate-active genes were identified using dbCAN2 (v2.0.11)

**Supplementary Table**

**Table S1 | Taxonomic affiliation and growth of the isolated strains on gellan gum in this study**. The growth of isolates was tested by two transfers on gellan gum as the sole substrate. Bacterial isolates that failed on growth test were not shown. Representative strains were depicted in bold.

| **Strain ID** | **Gelling agent** | **Inoc.** | **Temp.** | **Anaerobic conditions** | **Cysteine-HCl dose** | **Top-hit taxon** | **Top-hit strain** | **Similarity (%)** | **Growth on gellan gum** | **Growth on glucose** |
| --- | --- | --- | --- | --- | --- | --- | --- | --- | --- | --- |
| **G-JS-FE-0-2** | **gellan gum** | **JS** | **64℃** | **fermentation** | **0** | **Thermoanaerobacter sp.** | **strain X514** | **99.89%** | **F** | **T** |
| G-JS-FE-0-3 | gellan gum | JS | 64℃ | fermentation | 0 | Thermoanaerobacter sp. | strain X514 | 99.89% | F | T |
| G-JS-FE-0-7 | gellan gum | JS | 64℃ | fermentation | 0 | Thermoanaerobacter sp. | strain X514 | 99.89% | F | T |
| G-JS-FE-0-9a | gellan gum | JS | 64℃ | fermentation | 0 | Thermoanaerobacter sp. | strain X514 | 99.89% | F | T |
| **G-JS-FE-0-9b** | **gellan gum** | **JS** | **64℃** | **fermentation** | **0** | **Methanosarcina mazei** | **strain O1M9704b** | **99.35%** | **F** | **T** |
| G-JS-FE-0-11 | gellan gum | JS | 64℃ | fermentation | 0 | Thermoanaerobacter sp. | strain X514 | 99.89% | F | T |
| G-JS-FE-0.05-19 | gellan gum | JS | 64℃ | fermentation | 0.05 | Thermoanaerobacter sp. | strain X514 | 99.89% | F | T |
| G-JS-FE-0.05-21 | gellan gum | JS | 64℃ | fermentation | 0.05 | Thermoanaerobacter sp. | strain X514 | 99.89% | F | T |
| G-JS-FE-0.05-22a | gellan gum | JS | 64℃ | fermentation | 0.05 | Thermoanaerobacter sp. | strain X514 | 98.23% | F | T |
| G-JS-FE-0.05-22b | gellan gum | JS | 64℃ | fermentation | 0.05 | Methanosarcina sp. | MET5BHJ | 98.23% | F | T |
| G-JS-FE-0.05-25 | gellan gum | JS | 64℃ | fermentation | 0.05 | Thermoanaerobacter sp. | strain X514 | 99.89% | F | T |
| G-JS-FE-0.5-31 | gellan gum | JS | 64℃ | fermentation | 0.5 | Thermoanaerobacter sp. | strain X514 | 99.89% | F | T |
| G-JS-FE-0.5-33 | gellan gum | JS | 64℃ | fermentation | 0.5 | Thermoanaerobacter sp. | strain X514 | 99.89% | F | T |
| **G-JS-FE-0.5-35a** | **gellan gum** | **JS** | **64℃** | **fermentation** | **0.5** | **Acetomicrobium thermoterrnum** | **strain YWT-2** | **99.02%** | **F** | **T** |
| G-JS-FE-0.5-35b | gellan gum | JS | 64℃ | fermentation | 0.5 | Methanosarcina sp. | MET5BHJ | 98.23% | F | T |
| G-JS-FE-0.5-44 | gellan gum | JS | 64℃ | fermentation | 0.5 | Thermoanaerobacter sp. | strain X514 | 99.89% | F | T |
| G-JS-FE-0.5-47 | gellan gum | JS | 64℃ | fermentation | 0.5 | Thermoanaerobacter sp. | strain X514 | 99.89% | F | T |
| G-JS-FE-0.5-52 | gellan gum | JS | 64℃ | fermentation | 0.5 | Thermoanaerobacter sp. | strain X514 | 99.89% | F | T |
| G-JS-FE-0.5-53 | gellan gum | JS | 64℃ | fermentation | 0.5 | Acetomicrobium thermoterrnum | strain YWT-2 | 99.02% | F | T |
| G-JS-FE-0.5-64 | gellan gum | JS | 64℃ | fermentation | 0.5 | Thermoanaerobacter sp. | strain X514 | 99.89% | F | T |
| G-JS-FE-2-68 | gellan gum | JS | 64℃ | fermentation | 2 | Thermoanaerobacter sp. | strain X514 | 99.89% | F | T |
| G-JS-FE-2-73 | gellan gum | JS | 64℃ | fermentation | 2 | Thermoanaerobacter sp. | strain X514 | 99.89% | F | T |
| G-JS-FE-2-75 | gellan gum | JS | 64℃ | fermentation | 2 | Thermoanaerobacter sp. | strain X514 | 99.89% | F | T |
| G-JS-FE-2-77 | gellan gum | JS | 64℃ | fermentation | 2 | Thermoanaerobacter sp. | strain X514 | 99.89% | F | T |
| G-JS-FE-2-78 | gellan gum | JS | 64℃ | fermentation | 2 | Thermoanaerobacter sp. | strain X514 | 99.89% | F | T |
| G-JS-SR-0.5-1 | gellan gum | JS | 64℃ | sulfate reduction | 0.5 | Bacillus velezensis | strain KKLW | 100.00% | F | T |
| G-JS-SR-0.5-2 | gellan gum | JS | 64℃ | sulfate reduction | 0.5 | Bacillus velezensis | strain KKLW | 100.00% | F | T |
| G-JS-SR-0.5-3 | gellan gum | JS | 64℃ | sulfate reduction | 0.5 | Thermoanaerobacter pseudethanolicus | strain 9C | 97.28% | F | T |
| G-JS-SR-0.5-5 | gellan gum | JS | 64℃ | sulfate reduction | 0.5 | Thermoanaerobacter pseudethanolicus | strain 9C | 97.28% | F | T |
| G-JS-SR-0.5-7 | gellan gum | JS | 64℃ | sulfate reduction | 0.5 | Thermoanaerobacter pseudethanolicus | strain 9C | 97.28% | F | T |
| G-JS-SR-0.5-9 | gellan gum | JS | 64℃ | sulfate reduction | 0.5 | Thermoanaerobacter pseudethanolicus | strain 9C | 97.28% | F | T |
| G-JS-SR-0.5-10 | gellan gum | JS | 64℃ | sulfate reduction | 0.5 | Thermoanaerobacter pseudethanolicus | strain 9C | 97.28% | F | T |
| G-JS-SR-0.5-12 | gellan gum | JS | 64℃ | sulfate reduction | 0.5 | Bacillus velezensis | strain KKLW | 100.00% | F | T |
| G-JS-SR-0.5-13 | gellan gum | JS | 64℃ | sulfate reduction | 0.5 | Bacillus velezensis | strain KKLW | 100.00% | F | T |
| G-JS-SR-0.5-14 | gellan gum | JS | 64℃ | sulfate reduction | 0.5 | Thermoanaerobacter pseudethanolicus | strain 9C | 97.28% | F | T |
| G-JS-SR-0.5-15 | gellan gum | JS | 64℃ | sulfate reduction | 0.5 | Thermoanaerobacter pseudethanolicus | strain 9C | 97.28% | F | T |
| G-JS-SR-0.5-17 | gellan gum | JS | 64℃ | sulfate reduction | 0.5 | Thermoanaerobacter pseudethanolicus | strain 9C | 97.28% | F | T |
| G-JS-SR-2-18 | gellan gum | JS | 64℃ | sulfate reduction | 2 | Thermoanaerobacter pseudethanolicus | strain 9C | 97.28% | F | T |
| G-JS-SR-2-19 | gellan gum | JS | 64℃ | sulfate reduction | 2 | Bacillus velezensis | strain KKLW | 100.00% | T | T |
| G-JS-SR-2-20 | gellan gum | JS | 64℃ | sulfate reduction | 2 | Thermoanaerobacter pseudethanolicus | strain 9C | 97.28% | F | T |
| G-JS-SR-2-21 | gellan gum | JS | 64℃ | sulfate reduction | 2 | Thermoanaerobacter pseudethanolicus | strain 9C | 97.28% | F | T |
| G-JS-SR-2-22 | gellan gum | JS | 64℃ | sulfate reduction | 2 | Bacillus velezensis | strain KKLW | 100.00% | F | T |
| G-JS-SR-2-24 | gellan gum | JS | 64℃ | sulfate reduction | 2 | Thermoanaerobacter pseudethanolicus | strain 9C | 97.28% | F | T |
| G-JS-SR-2-26 | gellan gum | JS | 64℃ | sulfate reduction | 2 | Thermoanaerobacter pseudethanolicus | strain 9C | 97.28% | F | T |
| G-JS-SR-2-27 | gellan gum | JS | 64℃ | sulfate reduction | 2 | Thermoanaerobacter pseudethanolicus | strain 9C | 97.28% | F | T |
| G-JS-SR-2-28 | gellan gum | JS | 64℃ | sulfate reduction | 2 | Bacillus velezensis | strain KKLW | 100.00% | T | T |
| G-JS-SR-2-31 | gellan gum | JS | 64℃ | sulfate reduction | 2 | Thermoanaerobacter pseudethanolicus | strain 9C | 97.28% | F | T |
| G-JS-SR-2-32 | gellan gum | JS | 64℃ | sulfate reduction | 2 | Thermoanaerobacter pseudethanolicus | strain 9C | 97.28% | F | T |
| G-JS-SR-2-33 | gellan gum | JS | 64℃ | sulfate reduction | 2 | Bacillus velezensis | strain KKLW | 100.00% | F | T |
| G-JS-SR-2-35 | gellan gum | JS | 64℃ | sulfate reduction | 2 | Thermoanaerobacter pseudethanolicus | strain 9C | 97.28% | F | T |
| G-JS-SR-2-36 | gellan gum | JS | 64℃ | sulfate reduction | 2 | Thermoanaerobacter pseudethanolicus | strain 9C | 97.28% | F | T |
| G-JS-SR-2-38 | gellan gum | JS | 64℃ | sulfate reduction | 2 | Thermoanaerobacter pseudethanolicus | strain 9C | 97.28% | F | T |
| G-JS-SR-2-40 | gellan gum | JS | 64℃ | sulfate reduction | 2 | Bacillus velezensis | strain KKLW | 100.00% | T | T |
| G-JS-SR-5-41 | gellan gum | JS | 64℃ | sulfate reduction | 5 | Thermoanaerobacter pseudethanolicus | strain 9C | 97.28% | F | T |
| G-JS-SR-5-42 | gellan gum | JS | 64℃ | sulfate reduction | 5 | Thermoanaerobacter pseudethanolicus | strain 9C | 97.28% | F | T |
| G-JS-SR-5-43 | gellan gum | JS | 64℃ | sulfate reduction | 5 | Bacillus velezensis | strain KKLW | 100.00% | F | T |
| **G-JS-SR-5-44** | **gellan gum** | **JS** | **64℃** | **sulfate reduction** | **5** | **Bacillus subtilis** | **strain QZG5** | **99.34%** | **F** | **T** |
| G-JS-SR-5-46 | gellan gum | JS | 64℃ | sulfate reduction | 5 | Bacillus velezensis | strain KKLW | 100.00% | F | T |
| G-JS-SR-5-47 | gellan gum | JS | 64℃ | sulfate reduction | 5 | Bacillus velezensis | strain KKLW | 100.00% | F | T |
| G-JS-SR-5-48 | gellan gum | JS | 64℃ | sulfate reduction | 5 | Bacillus subtilis | strain QZG5 | 99.34% | F | T |
| G-JS-SR-5-49 | gellan gum | JS | 64℃ | sulfate reduction | 5 | Thermoanaerobacter pseudethanolicus | strain 9C | 97.28% | F | T |
| G-JS-SR-5-50 | gellan gum | JS | 64℃ | sulfate reduction | 5 | Thermoanaerobacter pseudethanolicus | strain 9C | 97.28% | F | T |
| G-JS-SR-5-51 | gellan gum | JS | 64℃ | sulfate reduction | 5 | Bacillus subtilis | strain QZG5 | 99.34% | F | T |
| G-JS-SR-5-52 | gellan gum | JS | 64℃ | sulfate reduction | 5 | Thermoanaerobacter pseudethanolicus | strain 9C | 97.28% | F | T |
| G-JS-SR-5-53 | gellan gum | JS | 64℃ | sulfate reduction | 5 | Thermoanaerobacter pseudethanolicus | strain 9C | 97.28% | F | T |
| G-JS-SR-5-55 | gellan gum | JS | 64℃ | sulfate reduction | 5 | Bacillus velezensis | strain KKLW | 100.00% | T | T |
| G-JS-SR-5-56 | gellan gum | JS | 64℃ | sulfate reduction | 5 | Bacillus subtilis | strain QZG5 | 99.34% | T | T |
| G-JS-SR-5-57 | gellan gum | JS | 64℃ | sulfate reduction | 5 | Thermoanaerobacter pseudethanolicus | strain 9C | 97.28% | F | T |
| G-JS-SR-5-58 | gellan gum | JS | 64℃ | sulfate reduction | 5 | Thermoanaerobacter pseudethanolicus | strain 9C | 97.28% | F | T |
| G-JS-SR-5-59 | gellan gum | JS | 64℃ | sulfate reduction | 5 | Bacillus velezensis | strain KKLW | 100.00% | F | T |
| G-JS-SR-5-60 | gellan gum | JS | 64℃ | sulfate reduction | 5 | Bacillus subtilis | strain QZG5 | 99.34% | F | T |
| **G-SL-SR-0.05-1** | **gellan gum** | **SL** | **55℃** | **sulfate reduction** | **0** | **Coprothermobacter proteolyticus** | **strain DSM 5265** | **99.60%** | **F** | **T** |
| **G-SL-SR-0-2** | **gellan gum** | **SL** | **55℃** | **sulfate reduction** | **0** | **Clostridium sartagoforme** | **strain CBA7517** | **99.58%** | **T** | **T** |
| G-SL-SR-0.05-7 | gellan gum | SL | 55℃ | sulfate reduction | 0 | Coprothermobacter proteolyticus | strain DSM 5265 | 99.60% | F | T |
| **G-SL-SR-0-9** | **gellan gum** | **SL** | **55℃** | **sulfate reduction** | **0** | **Thermatribacter velox** | **B11** | **97.41%** | **T** | **T** |
| **G-SL-SR-0-11** | **gellan gum** | **SL** | **55℃** | **sulfate reduction** | **0** | **Thermatribacter velox** | **B11** | **98.10%** | **T** | **T** |
| **G-SL-SR-0.05-14** | **gellan gum** | **SL** | **55℃** | **sulfate reduction** | **0** | **Thermodesulfovibrio yellowstonii** | **strain DSM 11347** | **99.30%** | **F** | **T** |
| G-SL-SR-0.05-15 | gellan gum | SL | 55℃ | sulfate reduction | 0 | Thermodesulfovibrio yellowstonii | strain DSM 11347 | 99.30% | F | T |
| G-SL-SR-0.05-16 | gellan gum | SL | 55℃ | sulfate reduction | 0 | Thermodesulfovibrio yellowstonii | strain DSM 11347 | 99.30% | F | T |
| G-SL-SR-0.05-17 | gellan gum | SL | 55℃ | sulfate reduction | 0 | Thermodesulfovibrio yellowstonii | strain DSM 11347 | 99.30% | F | T |
| G-SL-SR-0.05-18 | gellan gum | SL | 55℃ | sulfate reduction | 0 | Thermodesulfovibrio yellowstonii | strain DSM 11347 | 99.30% | F | T |
| G-SL-SR-0.05-19 | gellan gum | SL | 55℃ | sulfate reduction | 0 | Thermodesulfovibrio yellowstonii | strain DSM 11347 | 99.30% | F | T |
| **G-SL-SR-0.05-20** | **gellan gum** | **SL** | **55℃** | **sulfate reduction** | **0.05** | **Coprothermobacter proteolyticus** | **DSM 5265** | **98.64%** | **F** | **T** |
| G-SL-SR-0.05-28 | gellan gum | SL | 55℃ | sulfate reduction | 0.05 | Clostridium sartagoforme | strain CBA7517 | 99.58% | T | T |
| **G-SL-SR-0.05-29** | **gellan gum** | **SL** | **55℃** | **sulfate reduction** | **0.05** | **Atribacter laminatus** | **RT761** | **96.57%** | **T** | **T** |
| G-SL-SR-0.05-40 | gellan gum | SL | 55℃ | sulfate reduction | 0.05 | Thermatribacter velox | B11 | 98.10% | T | T |
| G-SL-SR-0.05-41 | gellan gum | SL | 55℃ | sulfate reduction | 0.05 | Thermodesulfovibrio yellowstonii | strain DSM 11347 | 99.30% | F | T |
| G-SL-SR-0.05-42 | gellan gum | SL | 55℃ | sulfate reduction | 0.05 | Thermodesulfovibrio yellowstonii | strain DSM 11347 | 99.30% | F | T |
| G-SL-SR-0.05-44 | gellan gum | SL | 55℃ | sulfate reduction | 0.05 | Thermodesulfovibrio yellowstonii | strain DSM 11347 | 99.30% | F | T |
| G-SL-SR-0.05-45 | gellan gum | SL | 55℃ | sulfate reduction | 0.05 | Thermodesulfovibrio yellowstonii | strain DSM 11347 | 99.30% | F | T |
| G-SL-SR-0.05-47 | gellan gum | SL | 55℃ | sulfate reduction | 0.05 | Thermodesulfovibrio yellowstonii | strain DSM 11347 | 99.30% | F | T |
| G-SL-SR-0.05-50 | gellan gum | SL | 55℃ | sulfate reduction | 0.05 | Coprothermobacter proteolyticus | DSM 5265 | 98.64% | F | T |
| G-SL-SR-0.05-52 | gellan gum | SL | 55℃ | sulfate reduction | 0.05 | Clostridium sartagoforme | strain CBA7517 | 99.58% | T | T |
| **G-SL-SR-0.05-53** | **gellan gum** | **SL** | **55℃** | **sulfate reduction** | **0.05** | **Atribacter laminatus** | **RT761** | **97.10%** | **T** | **T** |
| G-SL-SR-0.05-58 | gellan gum | SL | 55℃ | sulfate reduction | 0.05 | Thermatribacter velox | B11 | 98.10% | T | T |
| G-SL-SR-0.05-60 | gellan gum | SL | 55℃ | sulfate reduction | 0.05 | Thermodesulfovibrio yellowstonii | strain DSM 11347 | 99.30% | F | T |
| G-SL-SR-0.05-61 | gellan gum | SL | 55℃ | sulfate reduction | 0.05 | Thermodesulfovibrio yellowstonii | strain DSM 11347 | 99.30% | F | T |
| G-SL-SR-0.05-62 | gellan gum | SL | 55℃ | sulfate reduction | 0.05 | Thermodesulfovibrio yellowstonii | strain DSM 11347 | 99.30% | F | T |
| G-SL-SR-0.05-64 | gellan gum | SL | 55℃ | sulfate reduction | 0.05 | Thermodesulfovibrio yellowstonii | strain DSM 11347 | 99.30% | F | T |
| G-SL-SR-0.05-66 | gellan gum | SL | 55℃ | sulfate reduction | 0.05 | Thermodesulfovibrio yellowstonii | strain DSM 11347 | 99.30% | F | T |
| **G-SL-SR-0.5-67** | **gellan gum** | **SL** | **55℃** | **sulfate reduction** | **0.5** | **Clostridiales bacterium** | **strain MT110** | **98.53%** | **T** | **T** |
| G-SL-SR-0.5-68 | gellan gum | SL | 55℃ | sulfate reduction | 0.5 | Atribacter laminatus | RT761 | 97.10% | T | T |
| G-SL-SR-0.5-69 | gellan gum | SL | 55℃ | sulfate reduction | 0.5 | Thermodesulfovibrio yellowstonii | strain DSM 11347 | 99.30% | F | T |
| G-SL-SR-0.5-70 | gellan gum | SL | 55℃ | sulfate reduction | 0.5 | Thermodesulfovibrio yellowstonii | strain TGE-P1 | 99.30% | F | T |
| G-SL-SR-0.5-71 | gellan gum | SL | 55℃ | sulfate reduction | 0.5 | Coprothermobacter proteolyticus | DSM 5265 | 98.64% | F | T |
| G-SL-SR-0.5-75 | gellan gum | SL | 55℃ | sulfate reduction | 0.5 | Clostridiales bacterium | strain MT110 | 98.53% | T | T |
| G-SL-SR-0.5-76 | gellan gum | SL | 55℃ | sulfate reduction | 0.5 | Thermatribacter velox | B11 | 96.40% | T | T |
| G-SL-SR-0.5-77 | gellan gum | SL | 55℃ | sulfate reduction | 0.5 | Thermodesulfovibrio yellowstonii | strain DSM 11347 | 99.30% | F | T |
| G-SL-SR-0.5-78 | gellan gum | SL | 55℃ | sulfate reduction | 0.5 | Thermodesulfovibrio yellowstonii | strain TGE-P1 | 99.30% | F | T |
| G-SL-SR-0.5-79 | gellan gum | SL | 55℃ | sulfate reduction | 0.5 | Coprothermobacter proteolyticus | DSM 5265 | 98.64% | F | T |
| G-SL-SR-0.5-81 | gellan gum | SL | 55℃ | sulfate reduction | 0.5 | Clostridiales bacterium | strain MT110 | 98.53% | T | T |
| G-SL-SR-0.5-82 | gellan gum | SL | 55℃ | sulfate reduction | 0.5 | Thermatribacter velox | B11 | 96.40% | T | T |
| G-SL-SR-0.5-83 | gellan gum | SL | 55℃ | sulfate reduction | 0.5 | Thermodesulfovibrio yellowstonii | strain DSM 11347 | 99.30% | F | T |
| G-SL-SR-0.5-84 | gellan gum | SL | 55℃ | sulfate reduction | 0.5 | Thermodesulfovibrio yellowstonii | strain TGE-P1 | 99.30% | F | T |
| G-SL-SR-0.5-85 | gellan gum | SL | 55℃ | sulfate reduction | 0.5 | Coprothermobacter proteolyticus | DSM 5265 | 98.64% | F | T |
| G-SL-SR-0.5-86 | gellan gum | SL | 55℃ | sulfate reduction | 0.5 | Clostridiales bacterium | strain MT110 | 98.53% | T | T |
| G-SL-SR-0.5-87 | gellan gum | SL | 55℃ | sulfate reduction | 0.5 | Thermatribacter velox | B11 | 96.40% | T | T |
| G-SL-SR-0.5-88 | gellan gum | SL | 55℃ | sulfate reduction | 0.5 | Thermodesulfovibrio yellowstonii | strain DSM 11347 | 99.30% | F | T |
| G-SL-SR-0.5-89 | gellan gum | SL | 55℃ | sulfate reduction | 0.5 | Thermodesulfovibrio yellowstonii | strain TGE-P1 | 99.30% | F | T |
| G-SL-SR-0.5-90 | gellan gum | SL | 55℃ | sulfate reduction | 0.5 | Coprothermobacter proteolyticus | DSM 5265 | 98.64% | F | T |
| G-SL-SR-2-92 | gellan gum | SL | 55℃ | sulfate reduction | 2 | Thermatribacter velox | B11 | 96.40% | T | T |
| G-SL-SR-2-93 | gellan gum | SL | 55℃ | sulfate reduction | 2 | Thermatribacter velox | B11 | 96.40% | T | T |
| G-SL-SR-2-95 | gellan gum | SL | 55℃ | sulfate reduction | 2 | Thermatribacter velox | B11 | 96.40% | T | T |
| G-SL-SR-2-96 | gellan gum | SL | 55℃ | sulfate reduction | 2 | Thermatribacter velox | B11 | 96.40% | T | T |
| G-SL-SR-2-97 | gellan gum | SL | 55℃ | sulfate reduction | 2 | Thermatribacter velox | B11 | 96.40% | T | T |
| G-SL-SR-2-98 | gellan gum | SL | 55℃ | sulfate reduction | 2 | Thermatribacter velox | B11 | 96.40% | T | T |
| G-SL-FE-0.05-1 | gellan gum | SL | 55℃ | fermentation | 0 | Thermodesulfovibrio yellowstonii | strain DSM 11347 | 99.30% | F | T |
| G-SL-FE-0.05-2 | gellan gum | SL | 55℃ | fermentation | 0 | Thermodesulfovibrio yellowstonii | strain DSM 11347 | 99.30% | F | T |
| G-SL-FE-0.05-3 | gellan gum | SL | 55℃ | fermentation | 0 | Thermodesulfovibrio yellowstonii | strain DSM 11347 | 99.30% | F | T |
| G-SL-FE-0.05-5 | gellan gum | SL | 55℃ | fermentation | 0 | Thermodesulfovibrio yellowstonii | strain DSM 11347 | 99.30% | F | T |
| **G-SL-FE-0.5-6** | **gellan gum** | **SL** | **55℃** | **fermentation** | **0** | **Lacrimispora indolis** | **strain JCM 1380** | **97.94%** | **T** | **T** |
| G-SL-FE-0-7 | gellan gum | SL | 55℃ | fermentation | 0 | Thermatribacter velox | B11 | 96.40% | T | T |
| G-SL-FE-0.5-9 | gellan gum | SL | 55℃ | fermentation | 0 | Acetomicrobium thermoterrnum | strain YWT-2 | 99.02% | F | T |
| G-SL-FE-0.5-10 | gellan gum | SL | 55℃ | fermentation | 0 | Acetomicrobium thermoterrnum | strain YWT-2 | 99.02% | F | T |
| G-SL-FE-0.05-11 | gellan gum | SL | 55℃ | fermentation | 0 | Acetomicrobium thermoterrnum | strain YWT-2 | 99.02% | F | T |
| G-SL-FE-0-12 | gellan gum | SL | 55℃ | fermentation | 0 | Thermatribacter velox | B11 | 96.40% | T | T |
| G-SL-FE-0.5-13 | gellan gum | SL | 55℃ | fermentation | 0 | Thermodesulfovibrio yellowstonii | strain DSM 11347 | 99.30% | F | T |
| G-SL-FE-0.5-14 | gellan gum | SL | 55℃ | fermentation | 0 | Thermodesulfovibrio yellowstonii | strain DSM 11347 | 99.30% | F | T |
| G-SL-FE-0-15 | gellan gum | SL | 55℃ | fermentation | 0 | Lacrimispora indolis | strain JCM 1380 | 97.94% | T | T |
| G-SL-FE-0-16 | gellan gum | SL | 55℃ | fermentation | 0 | Thermatribacter velox | B11 | 96.40% | T | T |
| G-SL-FE-0-17 | gellan gum | SL | 55℃ | fermentation | 0 | Acetomicrobium thermoterrnum | strain YWT-2 | 99.02% | F | T |
| G-SL-FE-0.5-18 | gellan gum | SL | 55℃ | fermentation | 0 | Acetomicrobium thermoterrnum | strain YWT-2 | 99.02% | F | T |
| G-SL-FE-0.5-19 | gellan gum | SL | 55℃ | fermentation | 0 | Acetomicrobium thermoterrnum | strain YWT-2 | 99.02% | F | T |
| G-SL-FE-0-20 | gellan gum | SL | 55℃ | fermentation | 0 | Thermatribacter velox | B11 | 96.40% | T | T |
| G-SL-FE-0.05-21 | gellan gum | SL | 55℃ | fermentation | 0.05 | Thermatribacter velox | B11 | 96.40% | T | T |
| G-SL-FE-0.05-23 | gellan gum | SL | 55℃ | fermentation | 0.05 | Lacrimispora indolis | strain JCM 1380 | 97.94% | T | T |
| G-SL-FE-0.05-25 | gellan gum | SL | 55℃ | fermentation | 0.05 | Lacrimispora indolis | strain JCM 1380 | 97.94% | T | T |
| G-SL-FE-0.05-26 | gellan gum | SL | 55℃ | fermentation | 0.05 | Acetomicrobium thermoterrnum | strain YWT-2 | 99.02% | F | T |
| G-SL-FE-0.05-27 | gellan gum | SL | 55℃ | fermentation | 0.05 | Coprothermobacter proteolyticus | DSM 5265 | 98.64% | F | T |
| G-SL-FE-0.05-28 | gellan gum | SL | 55℃ | fermentation | 0.05 | Coprothermobacter proteolyticus | DSM 5265 | 98.64% | F | T |
| G-SL-FE-0.05-29 | gellan gum | SL | 55℃ | fermentation | 0.05 | Lacrimispora indolis | strain JCM 1380 | 97.94% | T | T |
| G-SL-FE-0.05-30 | gellan gum | SL | 55℃ | fermentation | 0.05 | Coprothermobacter proteolyticus | DSM 5265 | 98.64% | F | T |
| G-SL-FE-0.05-31 | gellan gum | SL | 55℃ | fermentation | 0.05 | Thermatribacter velox | B11 | 96.40% | T | T |
| G-SL-FE-0.05-32 | gellan gum | SL | 55℃ | fermentation | 0.05 | Lacrimispora indolis | strain JCM 1380 | 97.94% | T | T |
| G-SL-FE-0.05-34 | gellan gum | SL | 55℃ | fermentation | 0.05 | Lacrimispora indolis | strain JCM 1380 | 97.94% | T | T |
| G-SL-FE-0.05-35 | gellan gum | SL | 55℃ | fermentation | 0.05 | Acetomicrobium thermoterrnum | strain YWT-2 | 99.02% | F | T |
| G-SL-FE-0.05-36 | gellan gum | SL | 55℃ | fermentation | 0.05 | Coprothermobacter proteolyticus | DSM 5265 | 98.64% | F | T |
| G-SL-FE-0.05-37 | gellan gum | SL | 55℃ | fermentation | 0.05 | Coprothermobacter proteolyticus | DSM 5265 | 98.64% | F | T |
| G-SL-FE-0.05-39 | gellan gum | SL | 55℃ | fermentation | 0.05 | Lacrimispora indolis | strain JCM 1380 | 97.94% | T | T |
| G-SL-FE-0.05-40 | gellan gum | SL | 55℃ | fermentation | 0.05 | Coprothermobacter proteolyticus | DSM 5265 | 98.64% | F | T |
| G-SL-FE-0.5-41 | gellan gum | SL | 55℃ | fermentation | 0.5 | Coprothermobacter proteolyticus | DSM 5265 | 98.64% | F | T |
| G-SL-FE-0.5-44 | gellan gum | SL | 55℃ | fermentation | 0.5 | Coprothermobacter proteolyticus | DSM 5265 | 98.64% | F | T |
| G-SL-FE-0.5-45 | gellan gum | SL | 55℃ | fermentation | 0.5 | Thermatribacter velox | B11 | 97.41% | T | T |
| G-SL-FE-0.5-46 | gellan gum | SL | 55℃ | fermentation | 0.5 | Thermatribacter velox | B11 | 97.41% | T | T |
| G-SL-FE-0.5-47 | gellan gum | SL | 55℃ | fermentation | 0.5 | Thermatribacter velox | B11 | 97.41% | T | T |
| G-SL-FE-0.5-48 | gellan gum | SL | 55℃ | fermentation | 0.5 | Thermatribacter velox | B11 | 97.41% | T | T |
| G-SL-FE-0.5-49 | gellan gum | SL | 55℃ | fermentation | 0.5 | Thermatribacter velox | B11 | 97.41% | T | T |
| G-SL-FE-0.5-50 | gellan gum | SL | 55℃ | fermentation | 0.5 | Thermatribacter velox | B11 | 97.41% | T | T |
| G-SL-FE-0.5-51 | gellan gum | SL | 55℃ | fermentation | 0.5 | Thermatribacter velox | B11 | 97.41% | T | T |
| G-SL-FE-0.5-52 | gellan gum | SL | 55℃ | fermentation | 0.5 | Thermatribacter velox | B11 | 97.41% | T | T |
| G-SL-FE-0.5-53 | gellan gum | SL | 55℃ | fermentation | 0.5 | Coprothermobacter proteolyticus | DSM 5265 | 98.64% | F | T |
| G-SL-FE-0.5-54 | gellan gum | SL | 55℃ | fermentation | 0.5 | Coprothermobacter proteolyticus | DSM 5265 | 98.64% | F | T |
| G-SL-FE-0.5-55 | gellan gum | SL | 55℃ | fermentation | 0.5 | Thermatribacter velox | B11 | 97.41% | T | T |
| G-SL-FE-0.5-57 | gellan gum | SL | 55℃ | fermentation | 0.5 | Thermatribacter velox | B11 | 97.41% | T | T |
| G-SL-FE-0.5-58 | gellan gum | SL | 55℃ | fermentation | 0.5 | Thermatribacter velox | B11 | 97.41% | T | T |
| G-SL-FE-0.5-59 | gellan gum | SL | 55℃ | fermentation | 0.5 | Thermatribacter velox | B11 | 97.41% | T | T |
| G-SL-FE-2-63 | gellan gum | SL | 55℃ | fermentation | 2 | Acetomicrobium thermoterrnum | strain YWT-2 | 99.02% | F | T |
| G-SL-FE-2-64 | gellan gum | SL | 55℃ | fermentation | 2 | Acetomicrobium thermoterrnum | strain YWT-2 | 99.02% | F | T |
| G-SL-FE-2-65 | gellan gum | SL | 55℃ | fermentation | 2 | Thermatribacter velox | B11 | 97.41% | T | T |
| G-SL-FE-2-66 | gellan gum | SL | 55℃ | fermentation | 2 | Acetomicrobium thermoterrnum | strain YWT-2 | 99.02% | F | T |
| G-SL-FE-2-68 | gellan gum | SL | 55℃ | fermentation | 2 | Acetomicrobium thermoterrnum | strain YWT-2 | 99.02% | F | T |
| G-SL-FE-2-69 | gellan gum | SL | 55℃ | fermentation | 2 | Thermatribacter velox | B11 | 97.41% | T | T |
| G-SL-FE-2-70 | gellan gum | SL | 55℃ | fermentation | 2 | Acetomicrobium thermoterrnum | strain YWT-2 | 99.02% | F | T |
| G-SL-FE-2-72 | gellan gum | SL | 55℃ | fermentation | 2 | Acetomicrobium thermoterrnum | strain YWT-2 | 99.02% | F | T |
| G-SL-FE-2-74 | gellan gum | SL | 55℃ | fermentation | 2 | Thermatribacter velox | B11 | 97.41% | T | T |
| G-SL-FE-2-75 | gellan gum | SL | 55℃ | fermentation | 2 | Acetomicrobium thermoterrnum | strain YWT-2 | 99.02% | F | T |
| G-SL-FE-5-83 | gellan gum | SL | 55℃ | fermentation | 5 | Acetomicrobium thermoterrnum | strain YWT-2 | 99.02% | F | T |
| G-SL-FE-5-84 | gellan gum | SL | 55℃ | fermentation | 5 | Thermatribacter velox | B11 | 97.41% | T | T |
| G-SL-FE-2-85 | gellan gum | SL | 55℃ | fermentation | 5 | Acetomicrobium thermoterrnum | strain YWT-2 | 99.02% | F | T |
| G-SL-FE-2-90 | gellan gum | SL | 55℃ | fermentation | 5 | Acetomicrobium thermoterrnum | strain YWT-2 | 99.02% | F | T |
| G-SL-FE-5-91 | gellan gum | SL | 55℃ | fermentation | 5 | Acetomicrobium thermoterrnum | strain YWT-2 | 99.02% | F | T |
| G-SL-FE-5-94 | gellan gum | SL | 55℃ | fermentation | 5 | Thermatribacter velox | B11 | 96.40% | T | T |
| G-SL-FE-2-95 | gellan gum | SL | 55℃ | fermentation | 5 | Acetomicrobium thermoterrnum | strain YWT-2 | 99.02% | F | T |
| G-SL-NR-0.05-16 | gellan gum | SL | 55℃ | nitrate reduction | 0.05 | Thermodesulfovibrio yellowstonii | strain TGE-P1 | 99.30% | F | T |
| G-SL-NR-0.05-21 | gellan gum | SL | 55℃ | nitrate reduction | 0.05 | Thermodesulfovibrio yellowstonii | strain TGE-P1 | 99.30% | F | T |
| G-SL-NR-0.05-24 | gellan gum | SL | 55℃ | nitrate reduction | 0.05 | Thermodesulfovibrio yellowstonii | strain TGE-P1 | 99.30% | F | T |
| G-SL-NR-0.05-25 | gellan gum | SL | 55℃ | nitrate reduction | 0.05 | Thermodesulfovibrio yellowstonii | strain TGE-P1 | 99.30% | F | T |
| G-SL-NR-0.5-30 | gellan gum | SL | 55℃ | nitrate reduction | 0.5 | Thermodesulfovibrio yellowstonii | strain TGE-P1 | 99.30% | F | T |
| G-SL-NR-0.5-31 | gellan gum | SL | 55℃ | nitrate reduction | 0.5 | Thermodesulfovibrio yellowstonii | strain TGE-P1 | 99.30% | F | T |
| G-SL-NR-0.5-33 | gellan gum | SL | 55℃ | nitrate reduction | 0.5 | Thermodesulfovibrio yellowstonii | strain TGE-P1 | 99.30% | F | T |
| G-SL-NR-0.5-34 | gellan gum | SL | 55℃ | nitrate reduction | 0.5 | Thermodesulfovibrio yellowstonii | strain TGE-P1 | 99.30% | F | T |
| G-SL-NR-0.5-35 | gellan gum | SL | 55℃ | nitrate reduction | 0.5 | Thermodesulfovibrio yellowstonii | strain TGE-P1 | 99.30% | F | T |
| G-SL-NR-0.5-36 | gellan gum | SL | 55℃ | nitrate reduction | 0.5 | Thermodesulfovibrio yellowstonii | strain TGE-P1 | 99.30% | F | T |
| G-SL-NR-0.5-37 | gellan gum | SL | 55℃ | nitrate reduction | 0.5 | Thermodesulfovibrio yellowstonii | strain TGE-P1 | 99.30% | F | T |
| G-SL-NR-0.5-38 | gellan gum | SL | 55℃ | nitrate reduction | 0.5 | Thermodesulfovibrio yellowstonii | strain TGE-P1 | 99.30% | F | T |
| G-SL-NR-2-40 | gellan gum | SL | 55℃ | nitrate reduction | 2 | Thermodesulfovibrio yellowstonii | strain TGE-P1 | 99.30% | F | T |
| G-SL-NR-2-44 | gellan gum | SL | 55℃ | nitrate reduction | 2 | Thermodesulfovibrio yellowstonii | strain TGE-P1 | 99.30% | F | T |
| G-SL-NR-2-46 | gellan gum | SL | 55℃ | nitrate reduction | 2 | Thermodesulfovibrio yellowstonii | strain TGE-P1 | 99.30% | F | T |
| G-SL-NR-2-49 | gellan gum | SL | 55℃ | nitrate reduction | 2 | Thermodesulfovibrio yellowstonii | strain TGE-P1 | 99.30% | F | T |
| G-SL-NR-2-51 | gellan gum | SL | 55℃ | nitrate reduction | 2 | Thermodesulfovibrio yellowstonii | strain TGE-P1 | 99.30% | F | T |
| G-SL-NR-2-52 | gellan gum | SL | 55℃ | nitrate reduction | 2 | Thermodesulfovibrio yellowstonii | strain TGE-P1 | 99.30% | F | T |
| G-SL-NR-2-53 | gellan gum | SL | 55℃ | nitrate reduction | 2 | Thermodesulfovibrio yellowstonii | strain TGE-P1 | 99.30% | F | T |
| G-SL-NR-2-54 | gellan gum | SL | 55℃ | nitrate reduction | 2 | Thermodesulfovibrio yellowstonii | strain TGE-P1 | 99.30% | F | T |
| G-SL-NR-2-55 | gellan gum | SL | 55℃ | nitrate reduction | 2 | Thermodesulfovibrio yellowstonii | strain TGE-P1 | 99.30% | F | T |
| G-SL-NR-2-57 | gellan gum | SL | 55℃ | nitrate reduction | 2 | Thermodesulfovibrio yellowstonii | strain TGE-P1 | 99.30% | F | T |
| G-SL-NR-2-58 | gellan gum | SL | 55℃ | nitrate reduction | 2 | Thermodesulfovibrio yellowstonii | strain TGE-P1 | 99.30% | F | T |
| G-SL-NR-2-60 | gellan gum | SL | 55℃ | nitrate reduction | 2 | Thermodesulfovibrio yellowstonii | strain TGE-P1 | 99.30% | F | T |
| G-HK-FE-0.05-1 | gellan gum | HK | rt | fermentation | 0.05 | Bacillus velezensis | strain KKLW | 100.00% | F | T |
| G-HK-FE-0.05-3 | gellan gum | HK | rt | fermentation | 0.05 | Bacillus subtilis | strain QZG5 | 99.34% | F | T |
| G-HK-FE-0.05-4 | gellan gum | HK | rt | fermentation | 0.05 | Bacillus subtilis | strain QZG5 | 99.34% | F | T |
| G-HK-FE-0.05-5 | gellan gum | HK | rt | fermentation | 0.05 | Bacillus subtilis | strain QZG5 | 99.34% | F | T |
| G-HK-FE-0.05-7 | gellan gum | HK | rt | fermentation | 0.05 | Bacillus subtilis | strain QZG5 | 99.34% | F | T |
| G-HK-FE-0.05-8 | gellan gum | HK | rt | fermentation | 0.05 | Bacillus subtilis | strain QZG5 | 99.34% | F | T |
| G-HK-FE-0.5-9 | gellan gum | HK | rt | fermentation | 0.5 | Bacillus velezensis | strain KKLW | 100.00% | F | T |
| G-HK-FE-0.5-10 | gellan gum | HK | rt | fermentation | 0.5 | Bacillus subtilis | strain QZG5 | 99.34% | F | T |
| G-HK-FE-0.5-11 | gellan gum | HK | rt | fermentation | 0.5 | Bacillus velezensis | strain KKLW | 100.00% | F | T |
| G-HK-FE-0.5-12 | gellan gum | HK | rt | fermentation | 0.5 | Bacillus velezensis | strain KKLW | 100.00% | F | T |
| G-HK-FE-0.5-14 | gellan gum | HK | rt | fermentation | 0.5 | Bacillus velezensis | strain KKLW | 100.00% | F | T |
| G-HK-SR-0.5-1 | gellan gum | HK | rt | sulfate reduction | 0 | Bacillus velezensis | strain KKLW | 100.00% | F | T |
| G-HK-SR-0.5-2 | gellan gum | HK | rt | sulfate reduction | 0 | Bacillus velezensis | strain KKLW | 100.00% | F | T |
| G-HK-SR-2-3 | gellan gum | HK | rt | sulfate reduction | 0.5 | Bacillus subtilis | strain QZG5 | 99.34% | F | T |
| G-HK-SR-2-4 | gellan gum | HK | rt | sulfate reduction | 0.5 | Bacillus velezensis | strain KKLW | 100.00% | F | T |
| G-HK-SR-2-5 | gellan gum | HK | rt | sulfate reduction | 0.5 | Bacillus subtilis | strain QZG5 | 99.34% | F | T |
| G-HK-SR-5-6 | gellan gum | HK | rt | sulfate reduction | 5 | Bacillus velezensis | strain KKLW | 100.00% | F | T |
| G-HK-SR-5-7 | gellan gum | HK | rt | sulfate reduction | 5 | Bacillus subtilis | strain QZG5 | 99.34% | F | T |
| G-HK-SR-5-8 | gellan gum | HK | rt | sulfate reduction | 5 | Bacillus velezensis | strain KKLW | 100.00% | F | T |
| G-HK-NR-0.5-1 | gellan gum | HK | rt | nitrate reduction | 0 | Bacillus subtilis | strain QZG5 | 99.34% | F | T |
| G-HK-NR-0.5-2 | gellan gum | HK | rt | nitrate reduction | 0 | Bacillus subtilis | strain QZG5 | 99.34% | F | T |
| G-HK-NR-0.5-3 | gellan gum | HK | rt | nitrate reduction | 0 | Bacillus subtilis | strain QZG5 | 99.34% | F | T |
| G-HK-NR-0.5-4 | gellan gum | HK | rt | nitrate reduction | 0 | Bacillus subtilis | strain QZG5 | 99.34% | F | T |
| G-HK-NR-0.05-5 | gellan gum | HK | rt | nitrate reduction | 0.05 | Bacillus subtilis | strain QZG5 | 99.34% | F | T |
| G-HK-NR-0.05-6 | gellan gum | HK | rt | nitrate reduction | 0.05 | Bacillus subtilis | strain QZG5 | 99.34% | F | T |
| G-JS-FE-0-1 | gellan gum | JS | 64℃ | fermentation | 0 | Methanothermobacter sp. | THM-1 | 98.54% | F | F |
| G-JS-FE-0-5 | gellan gum | JS | 64℃ | fermentation | 0 | Methanosarcina sp. | MET5BHJ | 98.23% | F | F |
| G-JS-FE-0-12 | gellan gum | JS | 64℃ | fermentation | 0 | Methanothermobacter sp. | THM-1 | 98.54% | F | F |
| G-JS-FE-0-14 | gellan gum | JS | 64℃ | fermentation | 0 | Methanosarcina mazei | strain O1M9704b | 99.89% | F | F |
| G-JS-FE-0.05-23 | gellan gum | JS | 64℃ | fermentation | 0.05 | Methanosarcina mazei | strain O1M9704b | 99.35% | F | F |
| **G-JS-FE-0.05-24** | **gellan gum** | **JS** | **64℃** | **fermentation** | **0.05** | **Methanothermobacter thermophilus** | **strain DSM 6529** | **99.08%** | **F** | **F** |
| **G-JS-FE-0.05-26** | **gellan gum** | **JS** | **64℃** | **fermentation** | **0.05** | **Methanothermobacter sp.** | **THM-1** | **98.54%** | **F** | **F** |
| G-JS-FE-0.05-27 | gellan gum | JS | 64℃ | fermentation | 0.05 | Methanothermobacter thermophilus | strain DSM 6529 | 99.08% | F | F |
| G-JS-FE-0.05-28 | gellan gum | JS | 64℃ | fermentation | 0.05 | Methanothermobacter thermophilus | strain DSM 6529 | 99.08% | F | F |
| G-JS-FE-0.5-29 | gellan gum | JS | 64℃ | fermentation | 0.5 | Methanosarcina mazei | strain O1M9704b | 99.35% | F | F |
| G-JS-FE-0.5-38 | gellan gum | JS | 64℃ | fermentation | 0.5 | Methanosarcina mazei | strain O1M9704b | 99.35% | F | F |
| G-JS-FE-0.5-40 | gellan gum | JS | 64℃ | fermentation | 0.5 | Methanosarcina mazei | strain O1M9704b | 99.35% | F | F |
| G-JS-FE-0.5-42 | gellan gum | JS | 64℃ | fermentation | 0.5 | Methanothermobacter sp. | THM-1 | 98.54% | F | F |
| G-JS-FE-0.5-43 | gellan gum | JS | 64℃ | fermentation | 0.5 | Methanosarcina mazei | strain O1M9704b | 99.35% | F | F |
| G-JS-FE-0.5-46 | gellan gum | JS | 64℃ | fermentation | 0.5 | Methanosarcina mazei | strain O1M9704b | 99.35% | F | F |
| G-JS-FE-0.5-54 | gellan gum | JS | 64℃ | fermentation | 0.5 | Methanosarcina sp. | MET5BHJ | 98.23% | F | F |
| G-JS-FE-0.5-55 | gellan gum | JS | 64℃ | fermentation | 0.5 | Methanosarcina mazei | strain O1M9704b | 99.35% | F | F |
| G-JS-FE-0.5-60 | gellan gum | JS | 64℃ | fermentation | 0.5 | Methanosarcina mazei | strain O1M9704b | 99.35% | F | F |
| G-JS-FE-0.5-61 | gellan gum | JS | 64℃ | fermentation | 0.5 | Methanothermobacter sp. | THM-1 | 98.54% | F | F |
| G-JS-FE-0.5-63 | gellan gum | JS | 64℃ | fermentation | 0.5 | Methanosarcina mazei | strain O1M9704b | 99.35% | F | F |
| G-JS-FE-2-66 | gellan gum | JS | 64℃ | fermentation | 2 | Methanosarcina mazei | strain O1M9704b | 99.35% | F | F |
| G-JS-FE-2-67 | gellan gum | JS | 64℃ | fermentation | 2 | Methanosarcina mazei | strain O1M9704b | 99.35% | F | F |
| G-JS-FE-2-68 | gellan gum | JS | 64℃ | fermentation | 2 | Methanosarcina sp. | MET5BHJ | 98.23% | F | F |
| G-JS-FE-2-69 | gellan gum | JS | 64℃ | fermentation | 2 | Methanosarcina sp. | MET5BHJ | 98.23% | F | F |
| G-JS-FE-2-70 | gellan gum | JS | 64℃ | fermentation | 2 | Methanosarcina sp. | MET5BHJ | 98.23% | F | F |
| G-JS-FE-2-71 | gellan gum | JS | 64℃ | fermentation | 2 | Methanosarcina sp. | MET5BHJ | 98.23% | F | F |
| G-JS-FE-2-74 | gellan gum | JS | 64℃ | fermentation | 2 | Methanosarcina sp. | MET5BHJ | 98.23% | F | F |

**Table S2 | Taxonomic affiliation and growth of the isolated strains on agar/agarose in this study**. The growth of isolates was tested by two transfers on agar or agarose as the sole substrate. Bacterial isolates that failed on growth test were not shown. Representative strains were depicted in bold.

| **Strain ID** | **Gelling agent** | **Inoc.** | **Temp.** | **Anaerobic conditions** | **Cysteine-HCl concentration** | **Top-hit taxon** | **Top-hit strain** | **Similarity (%)** | **Growth on agar** | **Growth on galactose** |
| --- | --- | --- | --- | --- | --- | --- | --- | --- | --- | --- |
| A-JS-SR-0.5-42 | Agar | JS | 64℃ | sulfate reduction | 0.5 | Bacillus stercoris | strain D7XPN1 | 99.06% | T | T |
| A-JS-SR-0.5-2 | Agar | JS | 64℃ | sulfate reduction | 0.5 | Bacillus stercoris | strain D7XPN1 | 99.06% | T | T |
| A-JS-SR-0.05-17 | Agar | JS | 64℃ | sulfate reduction | 0.05 | Bacillus stercoris | strain D7XPN1 | 99.06% | T | T |
| A-JS-FE-0-21 | Agar | JS | 64℃ | fermentation | 0 | Bacillus stercoris | strain D7XPN1 | 99.06% | T | T |
| A-JS-FE-0.05-2 | Agar | JS | 64℃ | fermentation | 0.05 | Bacillus stercoris | strain D7XPN1 | 99.06% | T | T |
| A-JS-FE-0.05-29 | Agar | JS | 64℃ | fermentation | 0.05 | Bacillus stercoris | strain D7XPN1 | 99.06% | T | T |
| A-JS-FE-0.05-20 | Agar | JS | 64℃ | fermentation | 0.05 | Bacillus stercoris | strain D7XPN1 | 99.06% | T | T |
| A-JS-FE-0.05-1a | Agar | JS | 64℃ | fermentation | 0.05 | Bacillus stercoris | strain D7XPN1 | 99.06% | T | T |
| A-JS-FE-0.05-19a | Agar | JS | 64℃ | fermentation | 0.05 | Bacillus stercoris | strain D7XPN1 | 99.06% | T | T |
| A-JS-FE-0.05-14a | Agar | JS | 64℃ | fermentation | 0.05 | Bacillus stercoris | strain D7XPN1 | 99.06% | T | T |
| A-JS-FE-0.05-13a | Agar | JS | 64℃ | fermentation | 0.05 | Bacillus stercoris | strain D7XPN1 | 99.06% | T | T |
| A-JS-FE-0.05-10a | Agar | JS | 64℃ | fermentation | 0.05 | Bacillus stercoris | strain D7XPN1 | 99.06% | T | T |
| A-JS-SR-0.5-6 | Agar | JS | 64℃ | sulfate reduction | 0.5 | Bacillus stercoris | strain D7XPN1 | 99.06% | T | T |
| A-JS-SR-0.5-46 | Agar | JS | 64℃ | sulfate reduction | 0.5 | Bacillus stercoris | strain D7XPN1 | 99.06% | T | T |
| A-JS-FE-0-3 | Agar | JS | 64℃ | fermentation | 0 | Bacillus stercoris | strain D7XPN1 | 99.06% | T | T |
| A-JS-FE-0-17 | Agar | JS | 64℃ | fermentation | 0 | Bacillus stercoris | strain D7XPN1 | 99.06% | T | T |
| A-JS-SR-0.5-44 | Agar | JS | 64℃ | sulfate reduction | 0.5 | Bacillus stercoris | strain D7XPN1 | 99.06% | T | T |
| A-JS-SR-0.5-4 | Agar | JS | 64℃ | sulfate reduction | 0.5 | Bacillus stercoris | strain D7XPN1 | 99.06% | T | T |
| A-JS-SR-0.05-7 | Agar | JS | 64℃ | sulfate reduction | 0.05 | Bacillus stercoris | strain D7XPN1 | 99.06% | T | T |
| A-JS-FE-0-2 | Agar | JS | 64℃ | fermentation | 0 | Bacillus stercoris | strain D7XPN1 | 99.06% | T | T |
| A-JS-FE-0.05-35 | Agar | JS | 64℃ | fermentation | 0.05 | Bacillus stercoris | strain D7XPN1 | 99.06% | T | T |
| A-JS-FE-0.05-35 | Agar | JS | 64℃ | fermentation | 0.05 | Bacillus stercoris | strain D7XPN1 | 99.06% | T | T |
| A-JS-FE-0-37 | Agar | JS | 64℃ | fermentation | 0 | Bacillus stercoris | strain D7XPN1 | 99.06% | T | T |
| A-JS-FE-0-36 | Agar | JS | 64℃ | fermentation | 0 | Bacillus stercoris | strain D7XPN1 | 99.06% | T | T |
| A-JS-FE-0-16 | Agar | JS | 64℃ | fermentation | 0 | Bacillus stercoris | strain D7XPN1 | 99.06% | T | T |
| A-JS-FE-2-26 | Agar | JS | 64℃ | fermentation | 2 | Bacillus stercoris | strain D7XPN1 | 99.06% | T | T |
| A-JS-FE-2-18 | Agar | JS | 64℃ | fermentation | 2 | Bacillus stercoris | strain D7XPN1 | 99.06% | T | T |
| A-JS-FE-0-5 | Agar | JS | 64℃ | fermentation | 0 | Bacillus stercoris | strain D7XPN1 | 99.06% | T | T |
| A-JS-SR-2-4 | Agar | JS | 64℃ | sulfate reduction | 2 | Bacillus stercoris | strain D7XPN1 | 99.06% | T | T |
| A-JS-SR-2-1 | Agar | JS | 64℃ | sulfate reduction | 2 | Bacillus stercoris | strain D7XPN1 | 99.06% | T | T |
| A-SL-FE-0.5-4 | Agar | SL | 55℃ | fermentation | 0.5 | Thermodesulfovibrio yellowstonii | strain TGE-P1 | 99.30% | F | T |
| A-SL-FE-0.5-3 | Agar | SL | 55℃ | fermentation | 0.5 | Thermodesulfovibrio yellowstonii | strain DSM 11347 | 99.30% | F | T |
| A-SL-SR-2-45 | Agar | SL | 55℃ | sulfate reduction | 2 | Thermodesulfovibrio yellowstonii | strain DSM 11347 | 99.30% | F | T |
| A-SL-SR-2-41 | Agar | SL | 55℃ | sulfate reduction | 2 | Thermodesulfovibrio yellowstonii | strain DSM 11347 | 99.30% | F | T |
| A-SL-NR-0-26 | Agar | SL | 55℃ | nitrate reduction | 0 | Thermodesulfovibrio yellowstonii | strain TGE-P1 | 99.30% | F | T |
| A-SL-NR-0-24 | Agar | SL | 55℃ | nitrate reduction | 0 | Thermodesulfovibrio yellowstonii | strain DSM 11347 | 99.30% | F | T |
| A-SL-NR-0-16 | Agar | SL | 55℃ | nitrate reduction | 0 | Thermodesulfovibrio yellowstonii | strain DSM 11347 | 99.30% | F | T |
| A-SL-NR-0-14 | Agar | SL | 55℃ | nitrate reduction | 0 | Thermodesulfovibrio yellowstonii | strain TGE-P1 | 99.30% | F | T |
| A-JS-FE-0-18b | Agar | JS | 64℃ | fermentation | 0.05 | Methanosarcina mazei | JS-FE-0-9b | 99.86% | F | T |
| A-JS-FE-0-38b | Agar | JS | 64℃ | fermentation | 0.05 | Methanosarcina mazei | JS-FE-0-9b | 99.86% | F | T |
| A-JS-FE-0.05-34 | Agar | JS | 64℃ | fermentation | 0.05 | Methanosarcina mazei | strain O1M9704b | 99.60% | F | F |
| A-JS-FE-0.05-33 | Agar | JS | 64℃ | fermentation | 0.05 | Methanosarcina mazei | strain O1M9704b | 99.60% | F | F |
| A-JS-FE-0.05-32 | Agar | JS | 64℃ | fermentation | 0.05 | Methanosarcina mazei | zm-15 | 99.47% | F | F |
| A-JS-FE-0.05-31 | Agar | JS | 64℃ | fermentation | 0.05 | Methanosarcina mazei | zm-15 | 99.47% | F | F |
| A-JS-FE-0.05-39 | Agar | JS | 64℃ | fermentation | 0.05 | Methanosarcina mazei | strain O1M9704b | 99.34% | F | F |
| A-JS-FE-0.05-23 | Agar | JS | 64℃ | fermentation | 0.05 | Methanosarcina mazei | strain O1M9704b | 99.34% | F | F |
| **A-HK-FE-0.05-7** | **Agar** | **HK** | **rt** | **fermentation** | **0.05** | **Enterobacter hormaechei** | **strain DD3** | **99.79%** | **F** | **T** |
| A-SL-SR-0-17 | Agar | SL | 55℃ | sulfate reduction | 0 | Clostridium favososporum | strain DSM 5907 | 98.76% | T | T |
| A-SL-SR-0-15 | Agar | SL | 55℃ | sulfate reduction | 0 | Clostridium favososporum | strain DSM 5907 | 98.76% | T | T |
| A-SL-SR-0-13 | Agar | SL | 55℃ | sulfate reduction | 0 | Clostridium favososporum | strain DSM 5907 | 98.76% | T | T |
| A-SL-SR-0-10 | Agar | SL | 55℃ | sulfate reduction | 0 | Clostridium favososporum | strain DSM 5907 | 98.76% | T | T |
| A-SL-SR-0.5-25 | Agar | SL | 55℃ | sulfate reduction | 0.5 | Clostridium favososporum | strain DSM 5907 | 98.76% | T | T |
| A-SL-SR-0.5-24 | Agar | SL | 55℃ | sulfate reduction | 0.5 | Clostridium favososporum | strain DSM 5907 | 98.76% | T | T |
| A-SL-SR-0.05-19 | Agar | SL | 55℃ | sulfate reduction | 0.05 | Clostridium favososporum | strain DSM 5907 | 98.76% | T | T |
| A-SL-SR-0.05-17 | Agar | SL | 55℃ | sulfate reduction | 0.05 | Clostridium favososporum | strain DSM 5907 | 98.76% | T | T |
| A-SL-FE-0-2 | Agar | SL | 55℃ | fermentation | 0 | Clostridium favososporum | strain DSM 5907 | 98.76% | T | T |
| A-SL-FE-0-1 | Agar | SL | 55℃ | fermentation | 0 | Clostridium favososporum | strain DSM 5907 | 98.76% | T | T |
| A-SL-FE-0.05-8 | Agar | SL | 55℃ | fermentation | 0.05 | Clostridium favososporum | strain DSM 5907 | 98.76% | T | T |
| A-SL-SR-0-14 | Agar | SL | 55℃ | sulfate reduction | 0 | Clostridium indicum | strain PI-S10-A1B | 98.68% | T | T |
| A-SL-SR-0-12 | Agar | SL | 55℃ | sulfate reduction | 0 | Clostridium indicum | strain PI-S10-A1B | 98.68% | T | T |
| A-SL-SR-0.5-22 | Agar | SL | 55℃ | sulfate reduction | 0.5 | Clostridium indicum | strain PI-S10-A1B | 98.68% | T | T |
| A-SL-SR-0.5-21 | Agar | SL | 55℃ | sulfate reduction | 0.5 | Clostridium indicum | strain PI-S10-A1B | 98.68% | T | T |
| A-SL-SR-0.05-20 | Agar | SL | 55℃ | sulfate reduction | 0.05 | Clostridium indicum | strain PI-S10-A1B | 98.68% | T | T |
| A-SL-SR-0.05-18 | Agar | SL | 55℃ | sulfate reduction | 0.05 | Clostridium indicum | strain PI-S10-A1B | 98.68% | T | T |
| A-SL-SR-0.05-15 | Agar | SL | 55℃ | sulfate reduction | 0.05 | Clostridium indicum | strain PI-S10-A1B | 98.68% | T | T |
| A-SL-SR-0.05-13 | Agar | SL | 55℃ | sulfate reduction | 0.05 | Clostridium indicum | strain PI-S10-A1B | 98.68% | T | T |
| A-SL-FE-0-21 | Agar | SL | 55℃ | fermentation | 0 | Clostridium indicum | strain PI-S10-A1B | 98.68% | T | T |
| A-SL-FE-0.05-2 | Agar | SL | 55℃ | fermentation | 0.05 | Clostridium indicum | strain PI-S10-A1B | 98.68% | T | T |
| A-SL-FE-0-11 | Agar | SL | 55℃ | fermentation | 0 | Clostridium indicum | strain PI-S10-A1B | 98.71% | T | T |
| A-SL-FE-0.5-2 | Agar | SL | 55℃ | fermentation | 0.5 | Clostridium indicum | strain PI-S10-A1B | 98.71% | T | T |
| A-SL-FE-0.5-19 | Agar | SL | 55℃ | fermentation | 0.5 | Clostridium indicum | strain PI-S10-A1B | 98.71% | T | T |
| A-SL-FE-0.5-13 | Agar | SL | 55℃ | fermentation | 0.5 | Clostridium indicum | strain PI-S10-A1B | 98.71% | T | T |
| A-SL-FE-0.5-12 | Agar | SL | 55℃ | fermentation | 0.5 | Clostridium indicum | strain PI-S10-A1B | 98.71% | T | T |
| A-SL-FE-0.05-7 | Agar | SL | 55℃ | fermentation | 0.05 | Clostridium indicum | strain PI-S10-A1B | 98.71% | T | T |
| A-SL-FE-0.05-13 | Agar | SL | 55℃ | fermentation | 0.05 | Clostridium indicum | strain PI-S10-A1B | 98.71% | T | T |
| **A-HK-NR-0.05-11** | **Agar** | **HK** | **rt** | **nitrate reduction** | **0.05** | **Citrobacter freundii** | **strain Q. nilot** | **99.57%** | **F** | **T** |
| A-HK-NR-0.5-16 | Agar | HK | rt | nitrate reduction | 0.5 | Citrobacter freundii | strain Q. nilot | 99.79% | F | T |
| A-HK-FE-0.5-9 | Agar | HK | rt | fermentation | 0.5 | Bacillus velezensis | strain KKLW | 100.00% | T | T |
| A-HK-SR-0-6 | Agar | HK | rt | sulfate reduction | 0.05 | Bacillus velezensis | strain KKLW | 100.00% | T | T |
| A-HK-NR-0.5-15 | Agar | HK | rt | nitrate reduction | 0.5 | Bacillus velezensis | strain KKLW | 100.00% | T | T |
| A-HK-NR-0.5-13 | Agar | HK | rt | nitrate reduction | 0.5 | Bacillus velezensis | strain KKLW | 100.00% | T | T |
| A-HK-NR-0.05-12 | Agar | HK | rt | nitrate reduction | 0.05 | Bacillus velezensis | strain KKLW | 100.00% | T | T |
| A-HK-FE-0.05-8 | Agar | HK | rt | fermentation | 0.05 | Bacillus velezensis | strain KKLW | 100.00% | T | T |
| A-HK-SR-0-5 | Agar | HK | rt | sulfate reduction | 0 | Bacillus velezensis | strain KKLW | 100.00% | T | T |
| A-HK-SR-0-4 | Agar | HK | rt | sulfate reduction | 0 | Bacillus velezensis | strain KKLW | 100.00% | T | T |
| A-HK-SR-0-3 | Agar | HK | rt | sulfate reduction | 0 | Bacillus velezensis | strain KKLW | 100.00% | T | T |
| A-JS-FE-0-38a | Agar | JS | 64℃ | fermentation | 0 | Thermoanaerobacter | strain X514 | 99.63% | F | T |
| A-JS-FE-0-18a | Agar | JS | 64℃ | fermentation | 0 | Thermoanaerobacter | strain X514 | 99.63% | F | T |
| A-JS-SR-0.5-13 | Agar | JS | 64℃ | sulfate reduction | 0.5 | Thermoanaerobacter | strain X514 | 99.63% | F | T |
| A-JS-SR-0.5-12 | Agar | JS | 64℃ | sulfate reduction | 0.5 | Thermoanaerobacter | strain X514 | 99.63% | F | T |
| A-JS-SR-0.5-34 | Agar | JS | 64℃ | sulfate reduction | 0.5 | Thermoanaerobacter | strain X514 | 99.63% | F | T |
| A-JS-SR-0.5-3 | Agar | JS | 64℃ | sulfate reduction | 0.5 | Thermoanaerobacter | strain X514 | 99.63% | F | T |
| A-SL-FE-0.05-23 | Agar | SL | 55℃ | fermentation | 0.05 | Acetomicrobium thermoterrnum | strain YWT-2 | 99.02% | F | T |
| A-SL-FE-0.05-11 | Agar | SL | 55℃ | fermentation | 0.05 | Acetomicrobium thermoterrnum | strain YWT-2 | 99.02% | F | T |
| A-JS-SR-0.5-32 | Agar | JS | 64℃ | sulfate reduction | 0.5 | Acetomicrobium thermoterrnum | strain YWT-2 | 99.02% | F | T |
| A-JS-SR-0.5-22 | Agar | JS | 64℃ | sulfate reduction | 0.5 | Acetomicrobium thermoterrnum | strain YWT-2 | 99.02% | F | T |
| A-SL-NR-0.05-33 | Agar | SL | 55℃ | nitrate reduction | 0.05 | Acetomicrobium thermoterrnum | strain YWT-2 | 99.02% | F | T |
| A-SL-NR-0.05-24 | Agar | SL | 55℃ | nitrate reduction | 0.05 | Acetomicrobium thermoterrnum | strain YWT-2 | 99.02% | F | T |
| A-SL-NR-0.05-23 | Agar | SL | 55℃ | nitrate reduction | 0.05 | Acetomicrobium thermoterrnum | strain YWT-2 | 99.02% | F | T |
| A-SL-NR-0.05-22 | Agar | SL | 55℃ | nitrate reduction | 0.05 | Acetomicrobium thermoterrnum | strain YWT-2 | 99.02% | F | T |
| A-SL-NR-0.05-7 | Agar | SL | 55℃ | nitrate reduction | 0.05 | Acetomicrobium thermoterrnum | strain YWT-2 | 99.02% | F | T |
| A-SL-NR-0.05-25 | Agar | SL | 55℃ | nitrate reduction | 0.05 | Acetomicrobium thermoterrnum | strain YWT-2 | 99.02% | F | T |
| A-SL-NR-0.05-17 | Agar | SL | 55℃ | nitrate reduction | 0.05 | Acetomicrobium thermoterrnum | strain YWT-2 | 99.02% | F | T |
| A-SL-NR-0.05-15 | Agar | SL | 55℃ | nitrate reduction | 0.05 | Acetomicrobium thermoterrnum | strain YWT-2 | 99.02% | F | T |
| A-SL-SR-2-16 | Agar | SL | 55℃ | nitrate reduction | 2 | Acetomicrobium thermoterrnum | strain YWT-2 | 99.02% | F | T |
| A-SL-SR-2-11 | Agar | SL | 55℃ | nitrate reduction | 2 | Acetomicrobium thermoterrnum | strain YWT-2 | 99.02% | F | T |
| A-SL-FE-2-14 | Agar | SL | 55℃ | fermentation | 2 | Acetomicrobium thermoterrnum | strain YWT-2 | 99.02% | F | T |
| A-SL-FE-2-10 | Agar | SL | 55℃ | fermentation | 2 | Acetomicrobium thermoterrnum | strain YWT-2 | 99.02% | F | T |
| A-SL-NR-0-22 | Agar | SL | 55℃ | nitrate reduction | 0 | Acetomicrobium thermoterrnum | strain YWT-2 | 99.02% | F | T |
| A-SL-NR-0-20 | Agar | SL | 55℃ | nitrate reduction | 0 | Acetomicrobium thermoterrnum | strain YWT-2 | 99.02% | F | T |
| A-SL-NR-0-12 | Agar | SL | 55℃ | nitrate reduction | 0 | Acetomicrobium thermoterrnum | strain YWT-2 | 99.02% | F | T |
| A-SL-NR-0-11 | Agar | SL | 55℃ | nitrate reduction | 0 | Acetomicrobium thermoterrnum | strain YWT-2 | 99.02% | F | T |

**Table S3 | Growth test of representative strains on different substrates**. The growth of isolates was tested by two transfers on gellan, glucose, agar, galactose or L-Cysteine as the sole substrate.

| **Strain ID** | **Top-hit taxon** | **Top-hit strain** | **Growth on gellan** | **Growth on glucose** | **Growth on agar** | **Growth on galactose** | **Growth on L-Cysteine** |
| --- | --- | --- | --- | --- | --- | --- | --- |
| G-SL-SR-0.5-67 | Clostridiales bacterium | strain MT110 | T | T | T | T | T |
| G-SL-SR-0-2 | Clostridium sartagoforme | strain CBA7517 | T | T | F | - | T |
| G-SL-FE-0.5-6 | Lacrimispora indolis | strain JCM 1380 | T | T | F | - | T |
| G-JS-SR-5-44 | Bacillus subtilis | strain QZG5 | F | T | T | T | T |
| G-SL-SR-0.05-20 | Coprothermobacter proteolyticus | DSM 5265 | F | T | F | - | T |
| G-SL-SR-0.05-1 | Coprothermobacter proteolyticus | DSM 5265 | F | T | F | - | T |
| A-HK-NR-0.05-11 | Citrobacter freundii | strain Q. nilot | F | T | F | T | T |
| A-HK-FE-0.05-7 | Enterobacter hormaechei | strain DD3 | F | T | F | T | T |
| G-JS-FE-0-2 | Thermoanaerobacter sp. | strain X514 | F | T | F | T | F |
| G-JS-FE-0.5-35a | Acetomicrobium thermoterrnum | strain YWT-2 | F | T | F | T | T |
| G-SL-SR-0-9 | Thermatribacter velox | B11 | T | T | F | T | F |
| G-SL-SR-0-11 | Thermatribacter velox | B11 | T | T | F | T | F |
| G-SL-SR-0.05-14 | Thermodesulfovibrio yellowstonii | strain DSM 11347 | F | T | F | T | T |
| G-SL-SR-0.05-29 | Atribacter laminatus | RT761 | T | T | F | T | F |
| G-SL-SR-0.05-53 | Atribacter laminatus | RT761 | T | T | F | T | F |
| G-JS-FE-0-9b | Methanosarcina mazei | strain O1M9704b | F | F | F | F | F |
| G-JS-FE-0.05-24 | Methanothermobacter thermophilus | strain DSM 6529 | F | F | F | F | F |
| G-JS-FE-0.05-26 | Methanothermobacter sp. | THM-1 | F | F | F | F | F |

**Table S4 | Representative genomes retrieved from NCBI database.** Genome quality estimates were calculated using CheckM (v1.1.2).

| **Genome** | **Accession number** | **16S rRNA gene identity %** | **Completeness %** | **Contamination %** |
| --- | --- | --- | --- | --- |
| Thermoanaerobacter pseudethanolicus ATCC 33223 | GCA_000019085.1 | 99.86 | 99.52 | 0.24 |
| Bacillus stercoris strain D7XPN1 | GCA_029772985.1 | 99.06 | 98.28 | 6.58 |
| Lacrimispora indolis SR3 H204DRAFT | GCA_000421505.1 | 98.34 | 99.37 | 1.58 |
| Coprothermobacter proteolyticus strain DSM 5265 | GCF_000020945.1 | 99.60 | 100.00 | 0.00 |
| Thermodesulfovibrio yellowstonii DSM 11347 | GCF_000020985.1 | 99.30 | 99.08 | 3.67 |
| Clostridiales bacterium strain MT110 | GCA_015243575.1 | 98.53 | 98.58 | 1.06 |
| Acetomicrobium thermoterrenum DSM 13490 | GCA_900107215.1 | 98.81 | 100.00 | 0.00 |
| Clostridium sartagoforme strain DSM | GCF_000401215.1 | 99.60 | 98.52 | 1.86 |
| Citrobacter freundii 12681 | GCA_011064845.1 | 99.57 | 99.90 | 0.48 |
| Enterobacter quasihormaechei strain WCHEs120003 | GCA_004331385.1 | 99.47 | 99.97 | 0.25 |
| Atribacter sp. isolate AnMBR.bin213 | GCA_040769105.1 | 97.02 | 100.00 | 0.00 |
| Thermatribacter velox strain B11 | GCA_038396615.1 | 98.54 | 98.31 | 0.00 |
| Isolate G-SL-SR-0-11 | this study | 100.00 | 98.31 | 0.00 |

**Supplementary Figure**


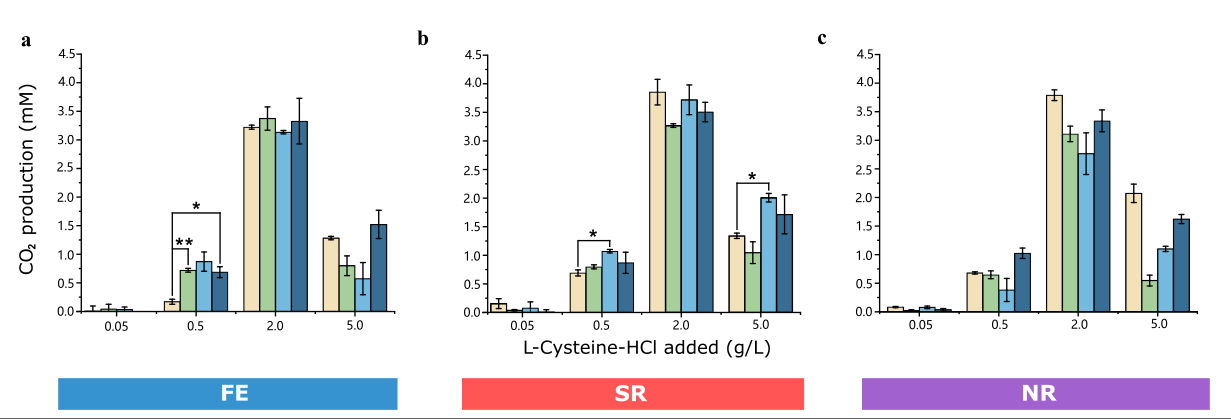


**FIG S1 Production of CO_2_ in enrichment cultures.** (**a-c**) Concentrations of CO_2_ in the headspace of enrichment cultures incubated under FE, SR and NR conditions. The samples of no-does L-Cys were not shown and the amounts of CO_2_ detected in these samples were subtracted from those of low-, regular- and high-does samples. Error bars represent standard errors. Cultures with significant differences in L-Cys or electron acceptors (sulfate and nitrate) consumption when compared with those in the control samples were indicated by an asterisk (**p* < 0.01 or **p* < 0.001).

**References**

1. Bankevich A, Nurk S, Antipov D, Gurevich A a., Dvorkin M, Kulikov AS, Lesin VM, Nikolenko SI, Pham S, Prjibelski AD, Pyshkin A V., Sirotkin A V., Vyahhi N, Tesler G, Alekseyev M a., Pevzner P a. 2012. SPAdes: A New Genome Assembly Algorithm and Its Applications to Single-Cell Sequencing. J Comput Biol 19:455–477.

2. Huerta-Cepas J, Szklarczyk D, Forslund K, Cook H, Heller D, Walter MC, Rattei T, Mende DR, Sunagawa S, Kuhn M, Jensen LJ, Von Mering C, Bork P. 2016. EGGNOG 4.5: A hierarchical orthology framework with improved functional annotations for eukaryotic, prokaryotic and viral sequences. Nucleic Acids Res 44:D286–D293.

3. Kanehisa M, Sato Y, Morishima K. 2016. BlastKOALA and GhostKOALA: KEGG Tools for Functional Characterization of Genome and Metagenome Sequences. J Mol Biol 428:726–731.

4. Aramaki T, Blanc-Mathieu R, Endo H, Ohkubo K, Kanehisa M, Goto S, Ogata H, Valencia A. 2020. KofamKOALA: KEGG Ortholog assignment based on profile HMM and adaptive score threshold. Bioinformatics 36:2251–2252.
